# Sustainable and Environmentally Friendly Approach for the Synthesis of Azoxybenzenes from the Reductive Dimerization of Nitrosobenzenes and the Oxidation of Anilines

**DOI:** 10.1021/acsomega.3c08328

**Published:** 2024-02-27

**Authors:** Idris Karakaya, Mehmet Mart, Ramazan Altundas

**Affiliations:** Department of Chemistry, College of Basic Sciences, Gebze Technical University, 41400 Gebze, Turkey

## Abstract

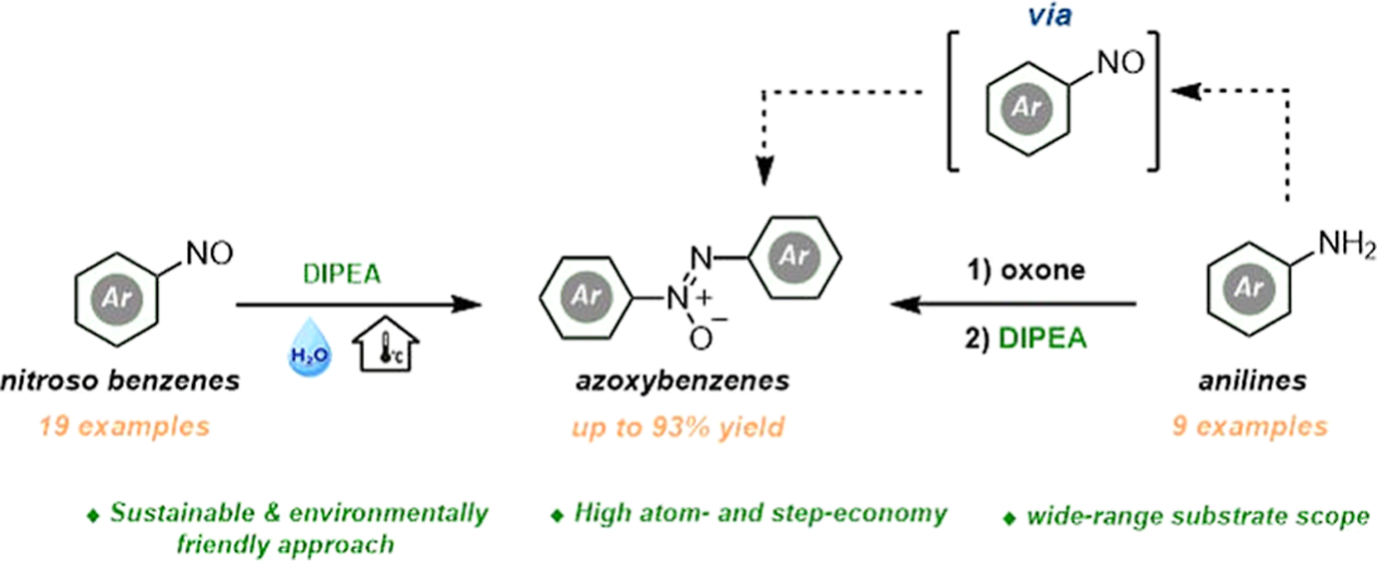

This study demonstrates a comparative synthesis of azoxybenzenes through the reductive dimerization of nitrosobenzenes and the oxidation of anilines. Utilizing the cost-effective DIPEA catalyst at room temperature with water as a green solvent, the one-pot procedure involves in situ generation of nitrosobenzene derivatives from anilines in the presence of oxone, followed by DIPEA addition. Both methods yield azoxybenzenes with high selectivity, showcasing the versatility of DIPEA in facilitating the synthesis of azoxybenzenes with various substituents in ortho, meta, and para positions, encompassing electron-donating and electron-withdrawing groups. The use of DIPEA proves pivotal in achieving moderate to high yields, emphasizing its significance in this environmentally friendly synthesis.

## Introduction

Azoxybenzenes are important synthons for synthetic organic chemistry due to their unique physical and chemical properties. Increasing interest has been observed both in the academic field and in the industrial sector, especially because azoxybenzenes can be used as dyes, reducing agents, chemical stabilizers, polymerization inhibitors, etc. In general, the synthesis of azoxybenzenes by the oxidation of anilines has been seen as a preferred approach because of the availability, stability, and relatively reasonable price of anilines.^1^

**Scheme 1:**
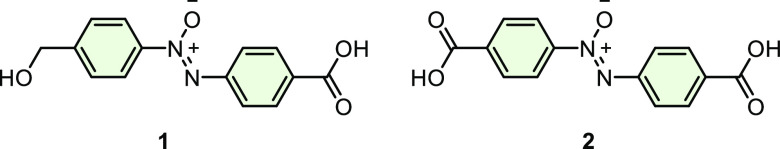
Examples of Natural Azoxybenzenes

In addition to being synthetically synthesized, azoxybenzenes are also found in the structure of some bacteria, fungi, plants, and sea sponges as a rare natural product group. Azoxy bonds make these structures capable of especially cytotoxic, nematocidal, and antimicrobial activities.^2^ For example, by isolating 4′-hydroxy-methylazoxybenzene-4-carboxylic acid **1** and azoxybenzene-4,4′-dicarboxylic acid **2** from the insect-parasitic fungus *Entomophthora virulenta*, their structures were elucidated, and it has been determined that it is actually the azoxybenzene structure that is responsible for the insecticidal activity of the parasitic fungus (Scheme 1).^3^

Academic interest against the synthesis of azoxybenzene from aniline, nitrobenzene, or nitrosobenzene derivatives is increasing day by day. Because of the availability, stability, and relatively reasonable price of anilines, the production of azoxybenzenes by direct oxidation of anilines has been seen as a preferred approach. For this reason, many oxidation methods using stoichiometric or catalytic systems have been reported.^4,5^

Considering the limited resources available and environmental pollution, scientific studies are encouraged to be carried out under green chemistry conditions. Green chemistry requires the development of new chemical reactivities and reaction conditions that can provide potential benefits for chemical syntheses in terms of resource and energy efficiency, product selectivity, operational simplicity, and health and environmental safety. In addition, green chemistry aims to discover new reactions that can maximize conversions of desired products and minimize byproducts, and to design new synthetic routes that can simplify processes in chemical production. It addresses such challenges by seeking out more environmentally friendly solvents and less energy demanding reaction conditions that are ecologically harmless in nature. However, most of the reported studies seem to be far from these standards, either because they generate excessive amounts of waste, because the solvents used are not environmentally friendly, or because they involve processes that require high energy. For this, it is necessary to abstain as much as possible from catalysts that require multistep synthesis and/or costly, expensive, and toxic solvents and reaction conditions that require high temperature or pressure. Instead, it becomes very attractive for industrial applications to perform chemical transformations without catalysts, if possible, or in the presence of commercially purchased or easily synthesized catalysts in one step, and in the presence of both inexpensive and environmentally friendly solvents such as water or ethanol, in ambient conditions that do not require any heating or pressure.^6,7^

**Scheme 2:**
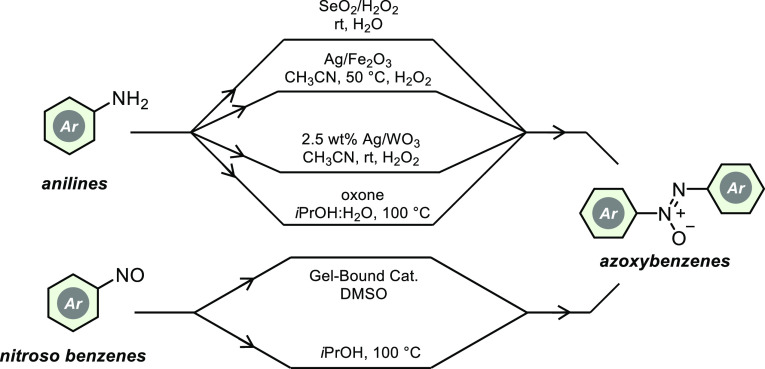
Some Reported Methods for the Synthesis of Azoxybenzenes

Many of the methods reported so far are burdened with one or more disadvantages, such as the use of toxic, harmful, and volatile organic solvents, the need for ligands that require multistep synthesis or the need for air-sensitive and expensive transition metal catalysts, high reaction temperatures, and/or long reaction times (Scheme 2).^5,7−9^

In this study, the synthesis of azoxybenzenes from nitrosobenzenes was obtained with high yields in the presence of commercially purchased cheap Hünig’s base (DIPEA) in a green, environmentally friendly, safe solvent, water, and at room temperature without the need for high-energy requirements. In addition, nitrosobenzene derivatives were formed in situ in a CH_3_CN/H_2_O mixed solvent system from aniline derivatives in the presence of oxone in a one-pot procedure, and then the target product synthesis was carried out with high product yields, again with the addition of DIPEA.

## Results and Discussions

To verify the applicability of the proposed protocol, the investigation was started by using nitrosobenzene (0.2 mmol) as the model substrate and DIPEA (2.0 equiv) as a catalyst under air at room temperature for 16 h. Then, several solvents were screened to determine the effect of the solvent on the desired transformation. While a moderate yield was obtained with DMSO, high yields were observed in the presence of the other solvents. The reason for the loss of yield in DMSO may be that the product cannot be sufficiently transferred from the aqueous phase to the organic phase during extraction. To our delight, an excellent yield of 93% was obtained when the reaction was carried out with water as the solvent. A series of screening reactions were performed in order to determine the effect of the catalyst amount, DIPEA, on the reaction course. Another result that pleased us was that the high yield was maintained even in the presence of only 0.25 equiv of DIPEA. As expected, no conversion was obtained from the control reaction, which was conducted without DIPEA. In the presence of 1.5 equiv of TEMPO, the reaction failed to yield any discernible products (entry 10). This indicates that the reaction proceeds through a radical mechanism.

**Scheme 3:**
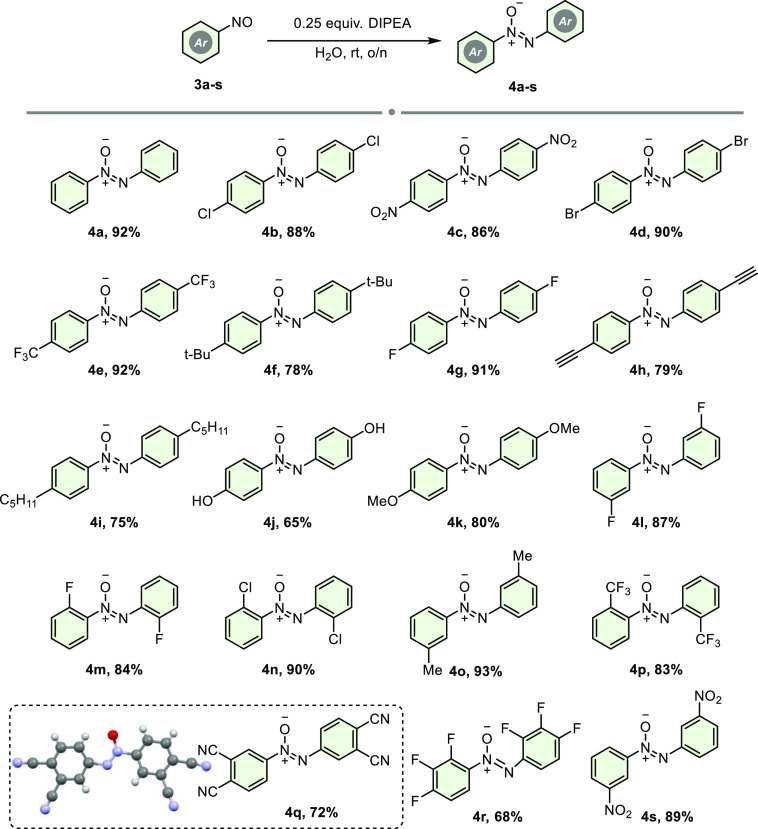
Reductive Dimerization of Nitrosobenzenes with DIPEA The reaction was performed with **3** (0.2 mmol) and DIPEA (0.25 equiv) in 2.0 mL of H_2_O for 16 h.

Having identified the optimal conditions, the reaction was afterward investigated using various nitrosobenzenes bearing different substitutions at the ortho, meta, and para positions, incorporating electron-donating and electron-withdrawing groups (Scheme 3). The corresponding products were acquired in moderate to good yields between 92 and 65%. Para-substituted sterically hindered groups *tert*-butyl and *n*-pentil containing nitrosobenzenes were converted to related azoxybenzenes with moderate yields of 78 and 75%, respectively. Nitrosobenzenes containing substituted F^–^, Cl^–^, and Br^–^ at different positions gave their target products high yields ranging from 84 to 91%. However, only a 68% yield was obtained from 1,2,3-trifluoro-4-nitrosobenzene. Azoxybenzene, which has a phthalonitrile structure, which is frequently used in the synthesis of phthalocyanine, was obtained in 72% yield, and its structure was fully elucidated by X-ray analysis.

**Scheme 4:**
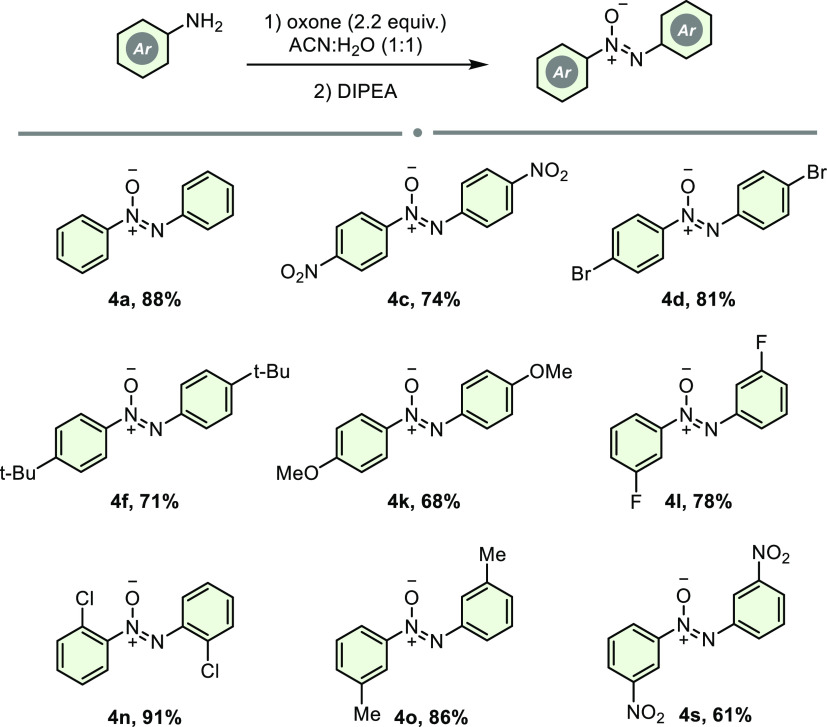
Azoxybenzenes from the Oxidation and Reductive Dimerization of Anilines The reaction was performed with **3** (0.2 mmol), oxone (2.2 equiv), and DIPEA (0.25 equiv) in 2.0 mL of ACN/H_2_O (1:1) for 16 h.

Nitrosobenzenes were obtained from the oxidation of anilines with oxone, following the literature.^10^ In order to demonstrate the feasibility and effectiveness of our method with the one-pot procedure, aniline derivatives were mixed in CH_3_CN/H_2_O (1:1) at room temperature by adding 2.2 equiv of oxone for about 1 h. Then, 0.25 equiv of DIPEA was added and allowed to mix overnight. Again, anilines were successfully converted to the corresponding azoxybenzene derivatives in moderate to good yields between 61 and 91% (Scheme 4). However, a slight decrease was observed compared with the reaction yields from the reductive dimerizations of nitrosobenzenes. This yield decline was probably due to the formation of byproducts such as diaza compounds or nitro compounds.^11^

**Scheme 5:**
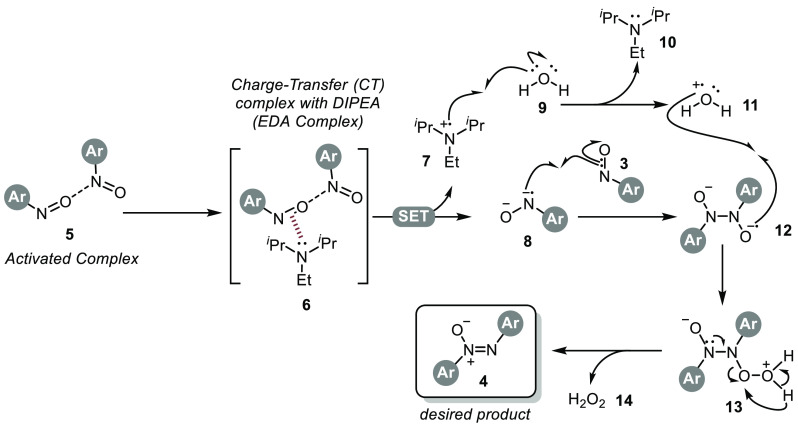
Plausible Mechanism

Based on the control experiment with TEMPO (Table 1, entry 10), we postulate that catalytic reductive dimerization of nitrosobenzenes takes place according to the radical pathway (Scheme 5). Chatterjee and his colleagues have suggested in their study that nitrosobenzene forms an active complex with allylsulfones, and this complex leads to the electrogeneration of radicals through the electron donor–acceptor (EDA) interaction with DIPEA.^12^ Similarly, our research indicates the generation of active complex **5** involving nitrosobenzenes. The reaction is expected to initiate with the transfer of an electron to nitrosobenzene, facilitated by the EDA complex **6** that forms between this complex and DIPEA. In the proposed mechanism, DIPEA interacts with the nitroso group, forming DIPEA radical **7** through a single electron transfer process. This interaction leads to the generation of the water radical **11**. Subsequently, radical water **11** and radical nitroso species **12** combine to form the **13** complex. In the final step, H_2_O_2_**14** is eliminated from the **13** complex, leading to the formation of the desired product **4**. Our control experiments and the proposed mechanism highlight the pivotal roles played by the catalyst DIPEA and water in guiding the progression of the reaction.

**Table 1:**
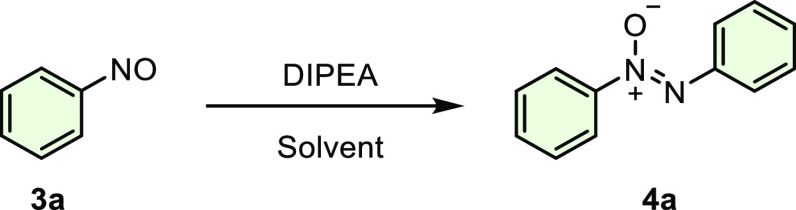
Comparison of Reaction Conditions^a^

entry	solvent	DIPEA (equiv)	yield (%)^b^
1	ACN	2.0	89
2	EtOH	2.0	95
3	H_2_O	2.0	93
4	MeOH	2.0	89
5	DMSO	2.0	71
6	H_2_O	1.0	94
7	H_2_O	0.5	89
8	H_2_O	0.25	92
9	H_2_O		n.r.
10^c^	ACN	0.25	n.r.

a The reaction was performed with **3a** (0.2 mmol) and DIPEA (*x* equiv) in 2.0 mL of solvent for 16 h.

b Isolated yields.

c Reaction was run in the presence of 1.5 equiv TEMPO.

In this research, DIPEA (*N*,*N*-diisopropylethylamine) emerges as a crucial catalyst, demonstrating its significance in guiding the environmentally friendly synthesis of azoxybenzenes. The versatility of DIPEA is highlighted in facilitating the high-yielding, selective synthesis of azoxybenzenes with diverse substituents. The proposed mechanism underscores DIPEA’s pivotal role in initiating radical pathways, emphasizing its instrumental contribution to the overall success of the catalytic reductive dimerization of nitrosobenzenes.

## Conclusions

In this study, an environmentally friendly, transition-metal-free, and economical new method, catalyzed by DIPEA, was developed for the synthesis of azoxybenzenes through the reductive dimerization of nitrosobenzenes and the oxidation of anilines. The fact that the described reaction takes place in the presence of both inexpensive and environmentally friendly solvents, such as water, and under ambient conditions that do not require heating or pressure makes our approach attractive for industrial applications. Due to the decrease in natural resources and high energy requirements, unconscious use, and increasing population, the tendency toward reactions that can be carried out with nature-friendly reagents and/or solvents and reaction conditions which do not require high energy is gradually increasing. The method we propose in this study requires milder conditions than all methods reported so far: an environmentally friendly solvent, room temperature, minimum energy, and using the commercial, cheap catalyst DIPEA.

## Experimental Section

### General Considerations

Unless otherwise noted, all substrates were purchased commercially and used without purification. NMR measurements were performed with a 500 MHz Bruker or 500 MHz Varian Mercury spectrometer. Single-crystal X-ray diffraction measurements were carried out on a Bruker APEX II CCD diffractometer.

### General Procedure to the Synthesis of Azoxybenzenes from the Dimerization of Nitrosobenzene

To a solution of nitrosobenzene (0.2 mmol, 21 mg) and DIPEA (0.05 mmol, 7 mg) in 2 mL of H_2_O was stirred at room temperature for 16 h. The reaction mixture was diluted with H_2_O (5 mL) and extracted with EtOAc (3 × 10 mL). The organic layers were combined and dried over MgSO_4_, filtered, and concentrated under reduced pressure. The crude product was purified by flash column chromatography eluting with EtOAc in hexanes, and the desired product was afforded in a yield of 92%.

### General Procedure to the Synthesis of Azoxybenzenes from One-Pot Oxidation–Reductive Dimerization of Anilines

To a solution of aniline (0.2 mmol, 17 mg) in CH_3_CN/H_2_O (1:1, 2.0 mL) was added oxone (2.2 equiv) and stirred for 2 h, then DIPEA (0.25 equiv) was added, and the reaction continued to stir at room temperature for 16 h. The reaction mixture was evaporated, diluted with H_2_O (5 mL), and extracted with EtOAc (3 × 10 mL). The organic layers were combined and dried over MgSO_4_, filtered, and concentrated under reduced pressure. The crude product was purified by flash column chromatography eluting with EtOAc in hexanes, and the desired product was afforded in a yield of 88%.

#### (*Z*)-1,2-Diphenyldiazene 1-Oxide (**4a**)^9^

^1^H NMR (CDCl_3_, 500 MHz): δ 8.34 (d, *J* = 6.9 Hz, 2H), 8.19 (d, *J* = 6.8 Hz, 2H), 7.64–7.46 (m, 5H), 7.42 (s, 1H).

#### (*Z*)-1,2-Bis(4-chlorophenyl)diazene 1-Oxide (**4b**)^9^

^1^H NMR (CDCl_3_, 500 MHz): δ 8.33–8.13 (m, 4H), 7.55–7.44 (m, 4H).

#### (*Z*)-1,2-Bis(4-nitrophenyl)diazene 1-Oxide (**4c**)^9^

^1^H NMR (CDCl_3_, 500 MHz): δ 8.54 (d, *J* = 9.2 Hz, 2H), 8.38 (d, *J* = 9.2 Hz, 4H), 8.30 (d, *J* = 9.2 Hz, 2H).

#### (*Z*)-1,2-Bis(4-bromophenyl)diazene 1-Oxide (**4d**)^9^

^1^H NMR (CDCl_3_, 500 MHz): δ 8.18 (d, *J* = 8.9 Hz, 2H), 8.08 (d, *J* = 8.8 Hz, 2H), 7.68–7.58 (m, 4H).

#### (*Z*)-1,2-Bis(4-(trifluoromethyl)phenyl)diazene 1-Oxide (**4e**)^13^

^1^H NMR (CDCl_3_, 500 MHz): δ 8.46 (d, *J* = 8.5 Hz, 2H), 8.23 (d, *J* = 8.4 Hz, 2H), 7.82 (d, *J* = 8.6 Hz, 2H), 7.76 (d, *J* = 8.5 Hz, 2H).

#### (*Z*)-1,2-Bis(4-(*tert*-butyl)phenyl)diazene 1-Oxide (**4f**)^14^

^1^H NMR (CDCl_3_, 500 MHz): δ 8.15 (d, *J* = 8.9 Hz, 4H), 7.53 (d, *J* = 8.9 Hz, 4H), 1.36 (s, 18H).

#### (*Z*)-1,2-Bis(4-fluorophenyl)diazene 1-Oxide (**4g**)^9^

^1^H NMR (CDCl_3_, 500 MHz): δ 8.36–8.29 (m, 2H), 8.29–8.23 (m, 2H), 7.22–7.13 (m, 4H).

#### (*Z*)-1,2-Bis(4-ethynylphenyl)diazene 1-Oxide (**4h**)^7^

^1^H NMR (CDCl_3_, 500 MHz): δ 8.28 (d, *J* = 8.8 Hz, 2H), 8.15 (d, *J* = 8.6 Hz, 2H), 7.61 (dd, *J* = 14.4, 8.6 Hz, 4H), 3.27 (s, 1H), 3.21 (s, 1H).

#### (*Z*)-1,2-Bis(4-pentylphenyl)diazene 1-Oxide (**4i**)^15^

^1^H NMR (CDCl_3_, 500 MHz): δ 8.14 (d, *J* = 8.6 Hz, 4H), 7.32 (d, *J* = 8.5 Hz, 4H), 2.73–2.68 (m, 4H), 1.68–1.62 (m, 4H), 1.37–1.31 (m, 8H), 0.90 (t, *J* = 6.9 Hz, 6H).

#### (*Z*)-1,2-Bis(4-hydroxyphenyl)diazene 1-Oxide (**4j**)^16^

^1^H NMR (CDCl_3_, 500 MHz): δ 8.17 (d, *J* = 9.1 Hz, 4H), 6.93 (d, *J* = 9.1 Hz, 4H), 6.22 (s, 2H).

#### (*Z*)-1,2-Bis(4-methoxyphenyl)diazene 1-Oxide (**4k**)^9^

^1^H NMR (CDCl_3_, 500 MHz): δ 8.22 (d, *J* = 9.3 Hz, 4H), 6.97 (d, *J* = 9.3 Hz, 4H), 3.92 (s, 6H).

#### (*Z*)-1,2-Bis(3-fluorophenyl)diazene 1-Oxide (**4l**)^17^

^1^H NMR (CDCl_3_, 500 MHz): δ 8.04 (d, *J* = 8.2 Hz, 1H), 8.00–7.94 (m, 2H), 7.77 (d, *J* = 8.1 Hz, 1H), 7.44–7.35 (m, 2H), 7.21 (td, *J* = 8.0, 2.2 Hz, 1H), 7.05 (td, *J* = 8.1, 2.2 Hz, 1H).

#### (*Z*)-1,2-Bis(2-fluorophenyl)diazene 1-Oxide (**4m**)^17^

^1^H NMR (CDCl_3_, 500 MHz): δ 8.29–8.26 (m, 1H), 7.96–7.89 (m, 1H), 7.54–7.50 (m, 1H), 7.41–7.35 (m, 1H), 7.30–7.27 (m, 2H), 7.25–7.19 (m, 2H).

#### (*Z*)-1,2-Bis(2-chlorophenyl)diazene 1-Oxide (**4n**)^7^

^1^H NMR (CDCl_3_, 500 MHz): δ 8.00 (dd, *J* = 8.0, 1.3 Hz, 1H), 7.76 (dd, *J* = 7.5, 1.9 Hz, 1H), 7.56–7.52 (m, 2H), 7.47–7.37 (m, 3H), 7.31 (td, *J* = 7.9, 1.4 Hz, 1H).

#### (*Z*)-1,2-Di-*m*-tolyldiazene 1-Oxide (**4o**)^18^

^1^H NMR (CDCl_3_, 500 MHz): δ 8.07 (d, *J* = 2.2 Hz, 1H), 7.98 (dd, *J* = 9.1, 2.6 Hz, 1H), 7.30–7.25 (m, 2H), 7.07 (d, *J* = 9.1 Hz, 1H), 7.03–6.97 (m, 3H), 2.37 (s, 3H), 2.33 (s, 3H).

#### (*Z*)-1,2-Bis(2-(trifluoromethyl)phenyl)diazene 1-Oxide (**4p**)^19^

^1^H NMR (CDCl_3_, 500 MHz): δ 8.02 (d, *J* = 8.1 Hz, 1H), 7.83–7.79 (m, 3H), 7.74 (t, *J* = 7.6 Hz, 1H), 7.67 (q, *J* = 7.9 Hz, 2H), 7.47 (t, *J* = 7.7 Hz, 1H).

#### (*Z*)-1,2-Bis(3,4-dicyanophenyl)diazene 1-Oxide (**4q**)

^1^H NMR (CDCl_3_, 500 MHz): δ 8.81 (d, *J* = 1.9 Hz, 1H), 8.72 (dd, *J* = 8.6, 2.1 Hz, 1H), 8.68 (d, *J* = 1.5 Hz, 1H), 8.45 (dd, *J* = 8.6, 1.7 Hz, 1H), 8.07 (d, *J* = 8.6 Hz, 1H), 7.97 (d, *J* = 8.6 Hz, 1H); ^13^C NMR (121 MHz, CDCl_3_): δ 149.65, 145.70, 139.30, 134.83, 134.45, 130.14, 130.02, 127.68, 127.04, 119.75, 117.56, 117.15, 116.56, 114.58, 114.02, 113.87.

#### (*Z*)-1,2-Bis(2,3,4-trifluorophenyl)diazene 1-Oxide (**4r**)^20^

^1^H NMR (CDCl_3_, 500 MHz): δ 8.33–8.25 (m, 1H), 7.85–7.78 (m, 1H), 7.19–7.04 (m, 2H).

#### (*Z*)-1,2-Bis(3-nitrophenyl)diazene 1-Oxide (**4s**)^9^

^1^H NMR (CDCl_3_, 500 MHz): δ 9.09 (t, *J* = 2.0 Hz, 1H), 8.59 (dd, *J* = 8.2, 2.1 Hz, 2H), 7.82 (t, *J* = 8.2 Hz, 1H).

## Data Availability

The data underlying this study are available in the published article and its online Supporting Information.

## Supporting Information Available

X-ray data and NMR spectral details of the synthesized compounds (PDF)
Crystallographic data for compund **4q** (CIF)

## Supplementary Material

ao3c08328_si_001.pdf

ao3c08328_si_002.cif
