# Supplementary material for: Sustainable and Environmentally Friendly Approach for the Synthesis of Azoxybenzenes from the Reductive Dimerization of Nitrosobenzenes and the Oxidation of Anilines
Source: ACS Omega. 2024 Feb 27;9(10):11494–9. doi: 10.1021/acsomega.3c08328 (PMC10938426; doi:10.1021/acsomega.3c08328)

Supporting Information

A sustainable and environmentally friendly approach for the synthesis of azoxybenzenes from the reductive dimerization of nitrosobenzenes and oxidation of anilines

Idris Karakaya,* Mehmet Mart, Ramazan Altundas

Department of Chemistry, College of Basic Sciences, Gebze Technical University, 41400 Gebze, Turkey

Email: karakaya@gtu.edu.tr

X-ray crystallographic data and structure refinement

Data for the single crystal compound were acquired with Bruker APEX II QUAZAR three-circle diffractometer. Crystal structure validations and geometrical calculations were applied using the Platon software. [1] Mercury software [2] was used for visualization of the .cif files. The structure (Figure S1) has been solved by the Bruker SHELXTL Software Package and refined using OLEX2.refine. [3, 4] The software used for molecular graphics: OLEX2 1.3 [5]; to prepare material for publication: OLEX2 1.3. [5] Data integration and reduction were realized with SAINT. [5] The crystallographic refinement data is summarized in Table S1.

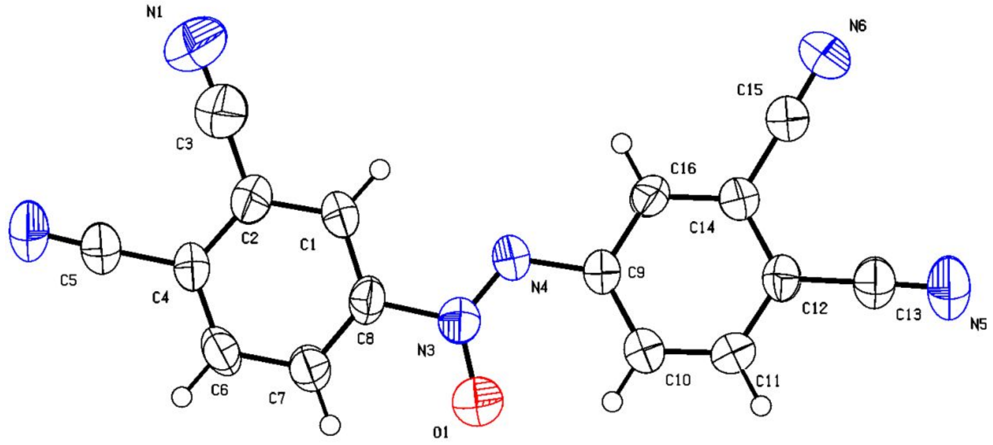

Figure S1: X-ray structure of compound **4q**

**Table S1:** Crystal data and structure refinement for **4q.**	
Identification code	23gtu144_IKA_452_2_0ma
Empirical formula	$C_{16}H_6N_6O$
Formula weight	298.27
Temperature/K	273.15
Crystal system	monoclinic
Space group	C2/c

a/Å	11.847(4)
b/Å	23.666(9)
c/Å	12.210(4)
α/°	90
β/°	112.565(5)
γ/°	90
Volume/Å^3	3161.4(18)
Z	8
ρ_{calc}g/cm^3	1.253
μ/mm^{-1}	0.085
F(000)	1216.0
Crystal size/mm^3	0.419 × 0.265 × 0.151
Radiation	MoKα (λ = 0.71073)
2Θ range for data collection/°	3.442 to 49.998
Index ranges	$-14 \le h \le 14, -28 \le k \le 28, -14 \le l \le 14$
Reflections collected	14347
Independent reflections	2784 [R_{int} = 0.0892, R_{sigma} = 0.0731]
Data/restraints/parameters	2784/0/208
Goodness-of-fit on F^2	1.258
Final R indexes [I>=2σ (I)]	R1 = 0.1124, wR2 = 0.3424
Final R indexes [all data]	R1 = 0.1721, wR2 = 0.3784
Largest diff. peak/hole / e Å^{-3}	0.94/-0.34

References

[1] A. L. Spek, Structure validation in chemical crystallography, Acta Crystallogr. D 65 (2009) 148–155.
[2] C. F. Macrae, P. R. Edgington, P. McCabe, E. Pidcock, G. P. Shields, R. Taylor, M. Towler , J. van De Streek, Mercury: visualization and analysis of crystal structures, J. Appl. Crystallogr. 39 (2006) 453–457.
[3] L. J. Bourhis, O. V. Dolomanov, R. J. Gildea, J. A. K. Howard, H. Puschmann, The anatomy of a comprehensive constrained, restrained refinement program for the modern computing environment-Olex2 dissected, Acta Crystallographica a-Foundat. Adv. 71 (2015) 59–75.
[4] G. M. Sheldrick, SHELXT - integrated space-group and crystal-structure deter- mination, Acta Crystallographica a-Foundat. Adv. 71 (2015) 3–8.
[5] SAINT, Version 8.34A, Bruker (2013).

Spectra

^{1}H NMR ($CDCl_3$, 500 MHz) spectrum of *(Z)*-1,2-diphenyldiazene 1-oxide (**4a**)

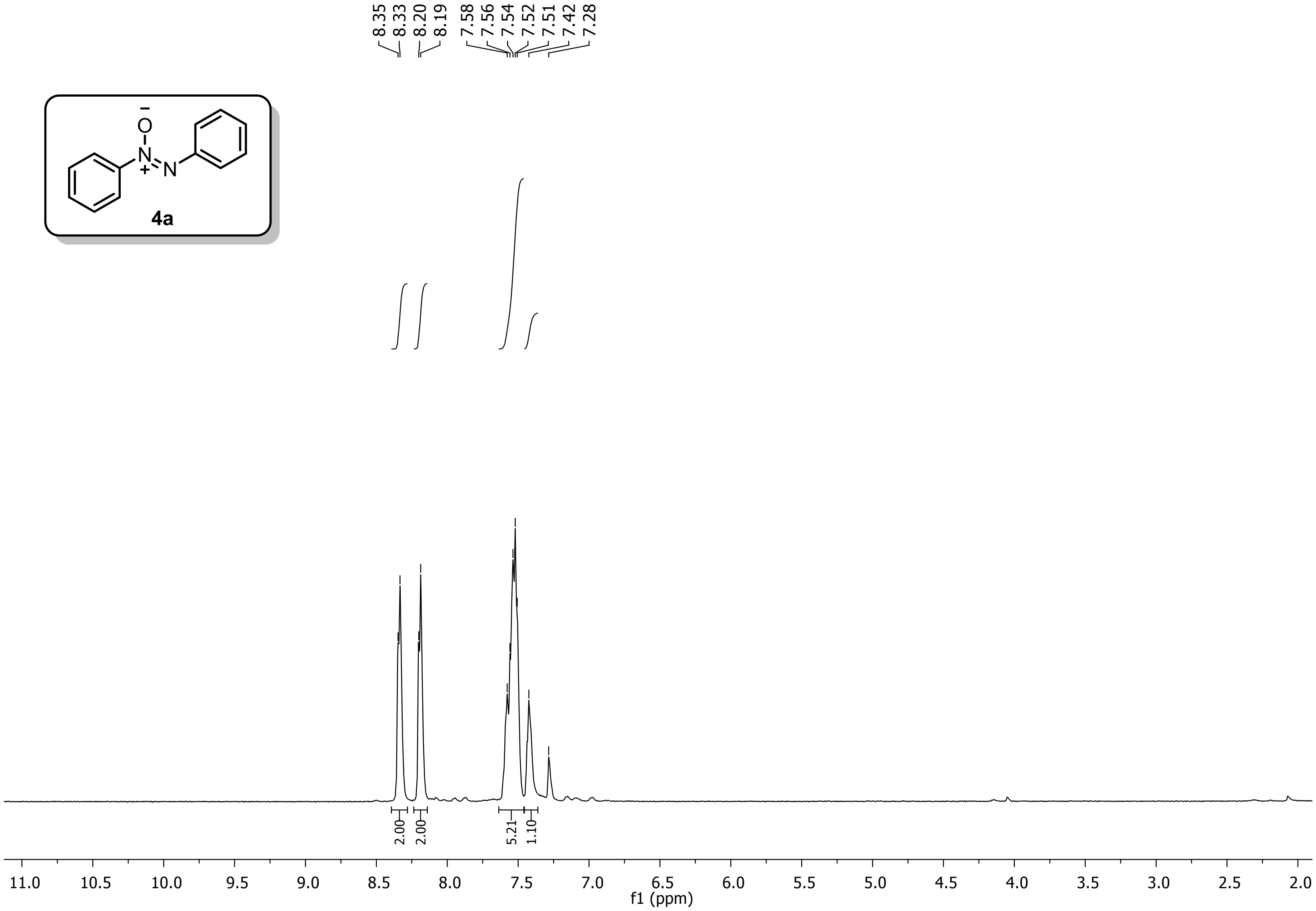

^{1}H NMR ($CDCl_3$, 500 MHz) spectrum of (*Z*)-1,2-bis(4-chlorophenyl)diazene 1-oxide (**4b**)

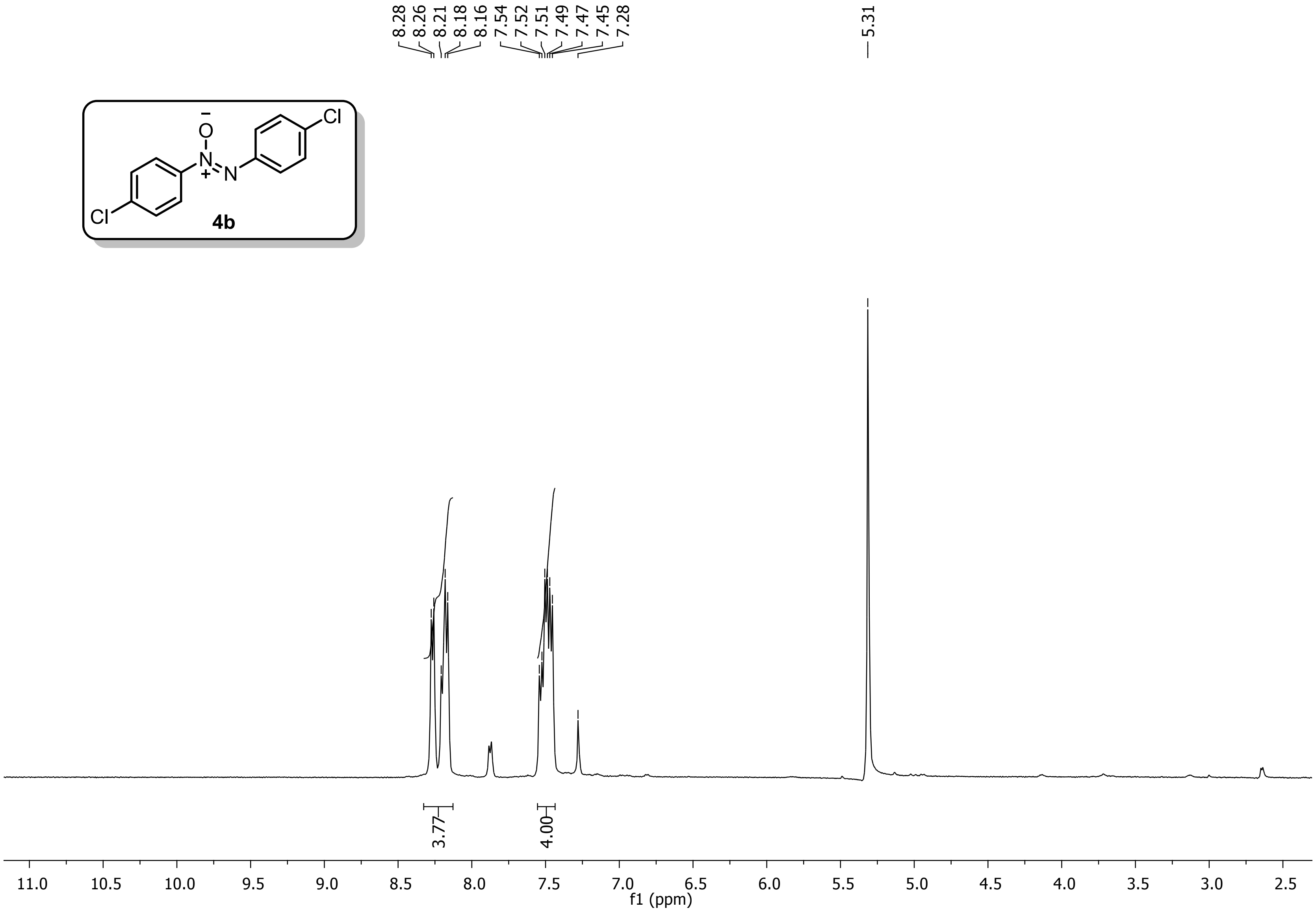

^{1}H NMR ($CDCl_3$, 500 MHz) spectrum of (*Z*)-1,2-bis(4-nitrophenyl)diazene 1-oxide (**4c**)

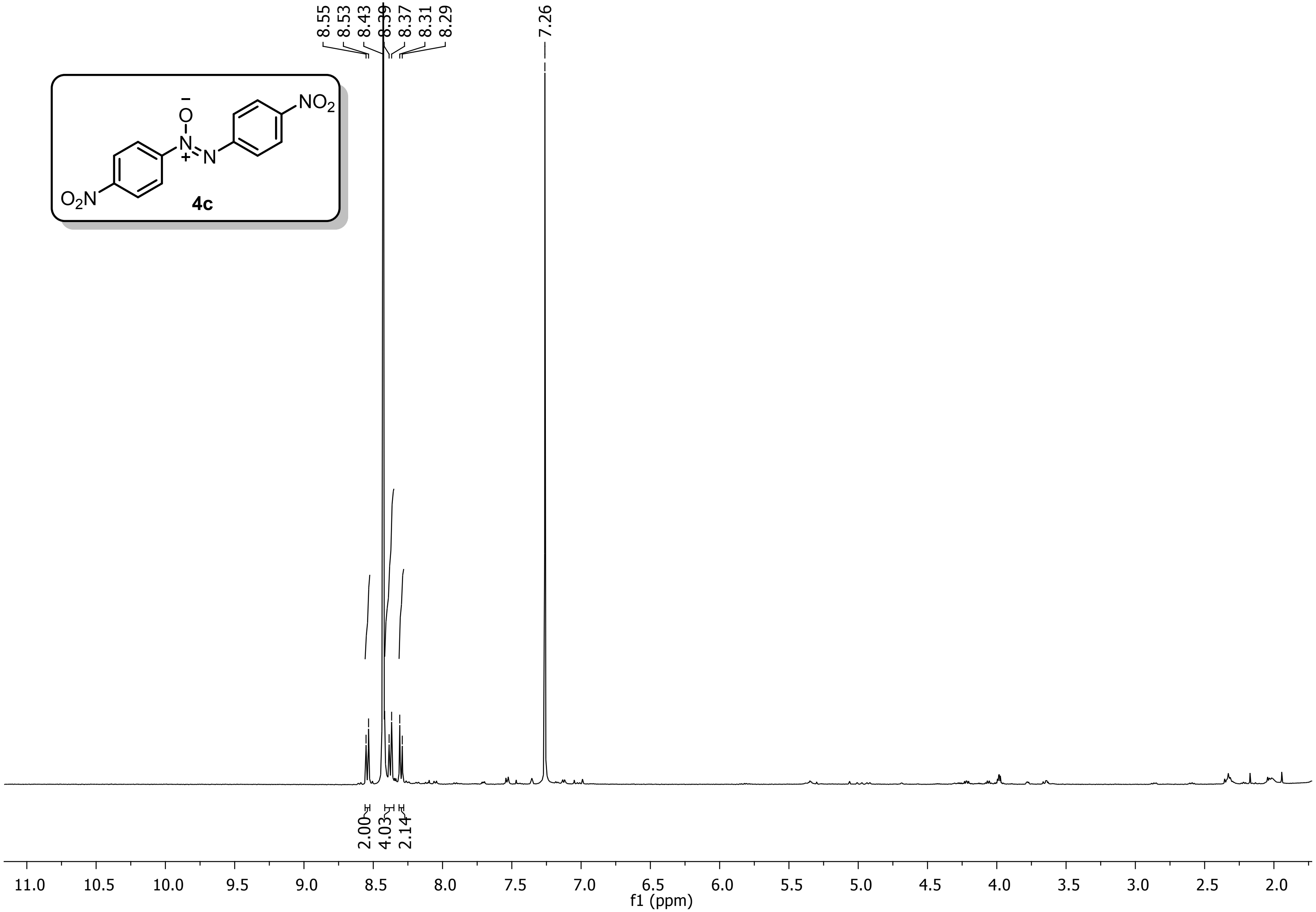

^{1}H NMR ($CDCl_3$, 500 MHz) spectrum of (*Z*)-1,2-bis(4-bromophenyl)diazene 1-oxide (**4d**)

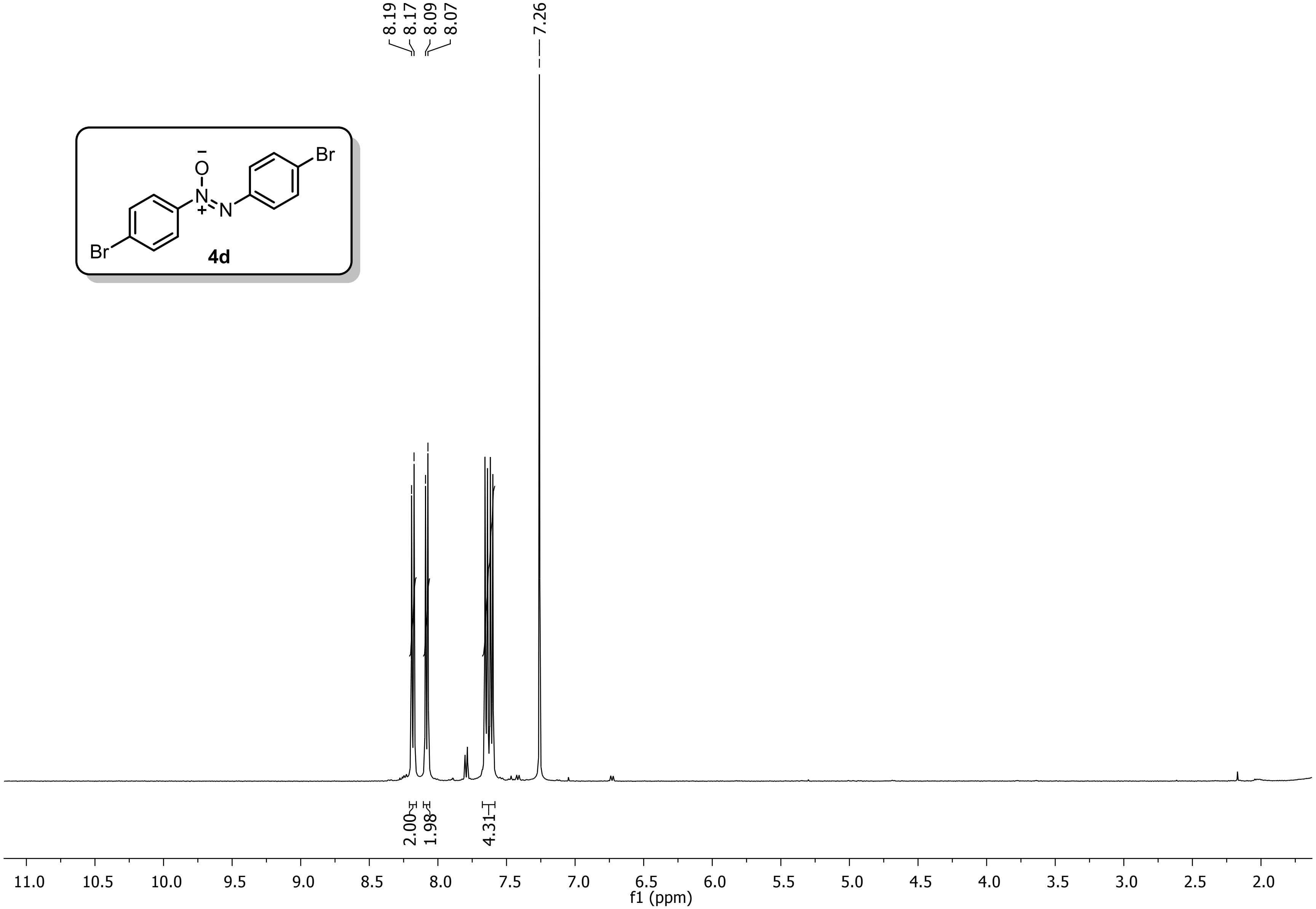

^{1}H NMR ($CDCl_3$, 500 MHz) spectrum of (*Z*)-1,2-bis(4-(trifluoromethyl)phenyl)diazene 1-oxide (**4e**)

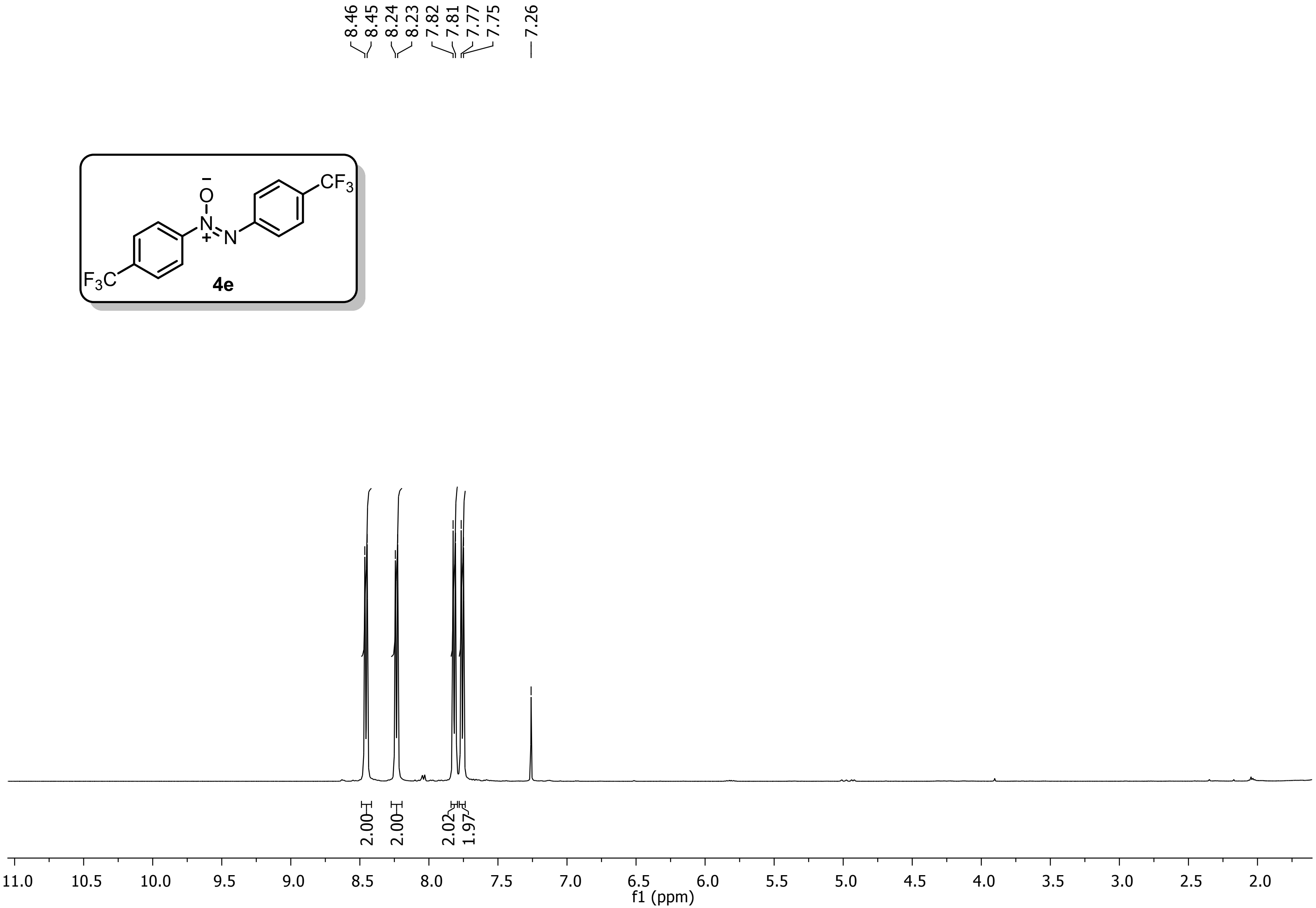

VX

^{1}H NMR ($CDCl_3$, 500 MHz) spectrum of (*Z*)-1,2-bis(4-(*tert*-butyl)phenyl)diazene 1-oxide (**4f**)

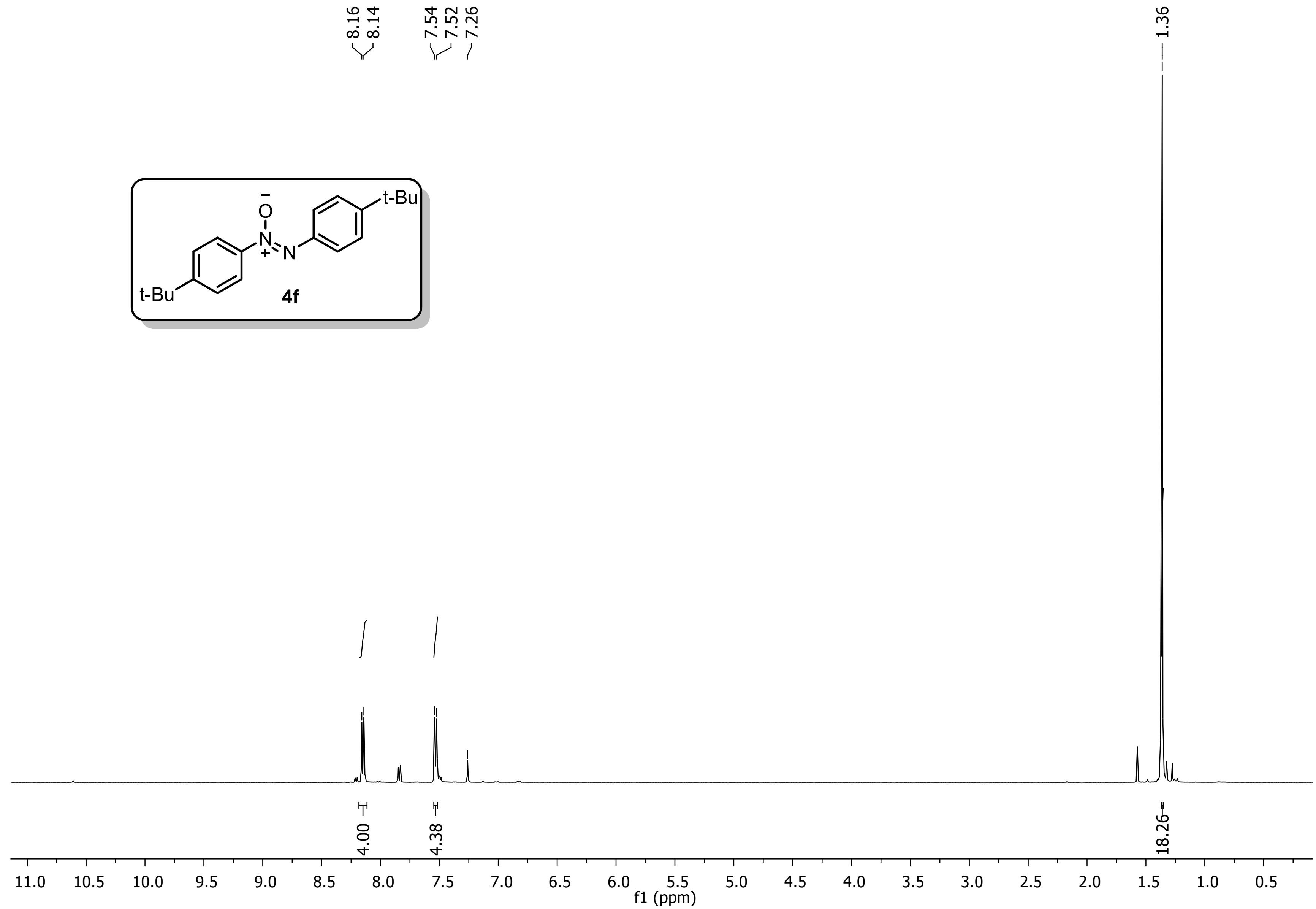

^{1}H NMR ($CDCl_3$, 500 MHz) spectrum of (*Z*)-1,2-bis(4-fluorophenyl)diazene 1-oxide (**4g**)

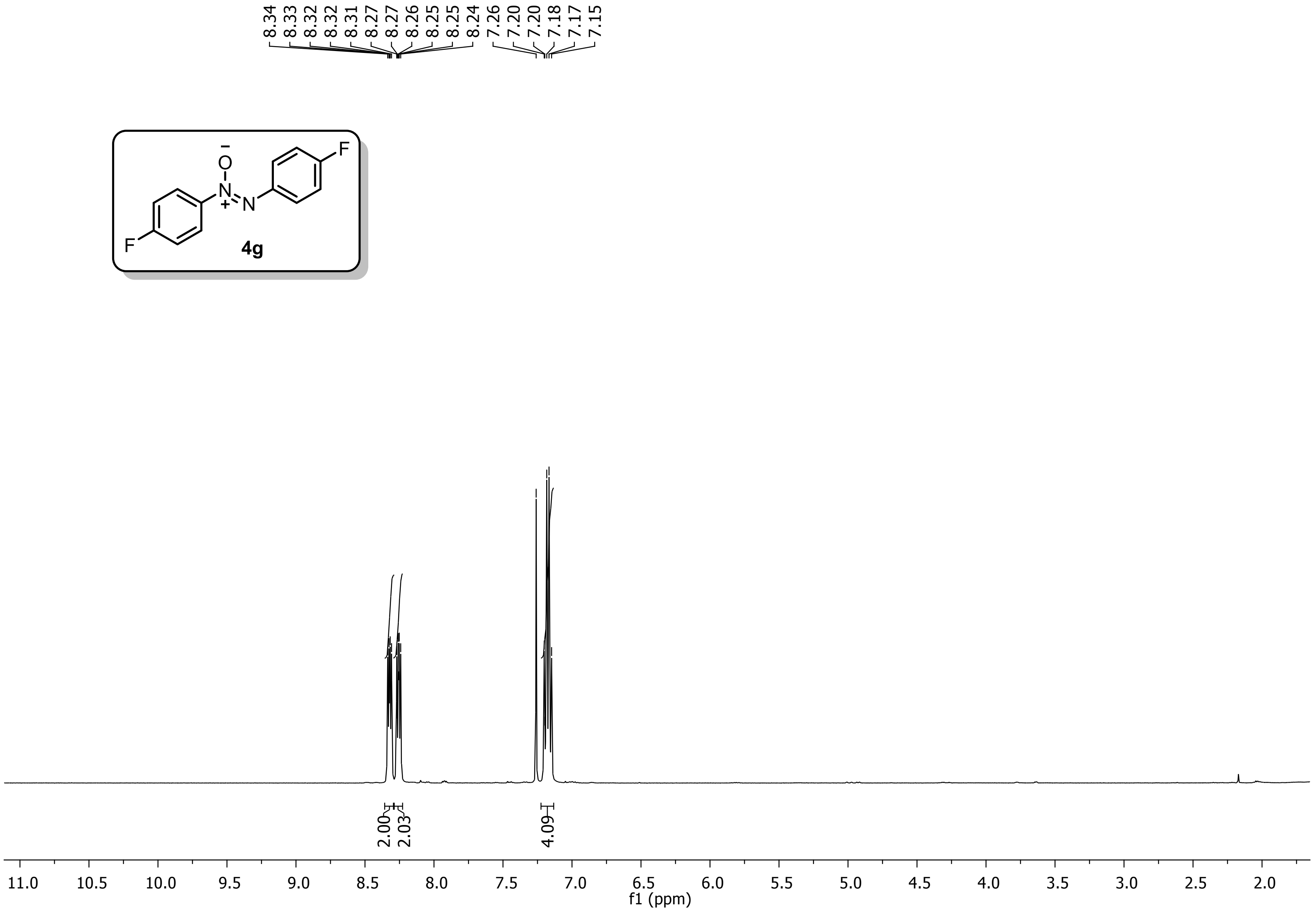

^{1}H NMR ($CDCl_3$, 500 MHz) spectrum of (*Z*)-1,2-bis(4-ethynylphenyl)diazene 1-oxide (**4h**)

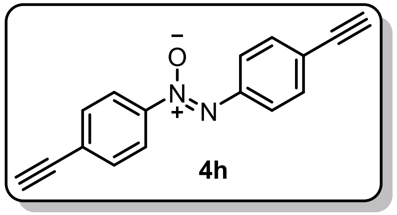

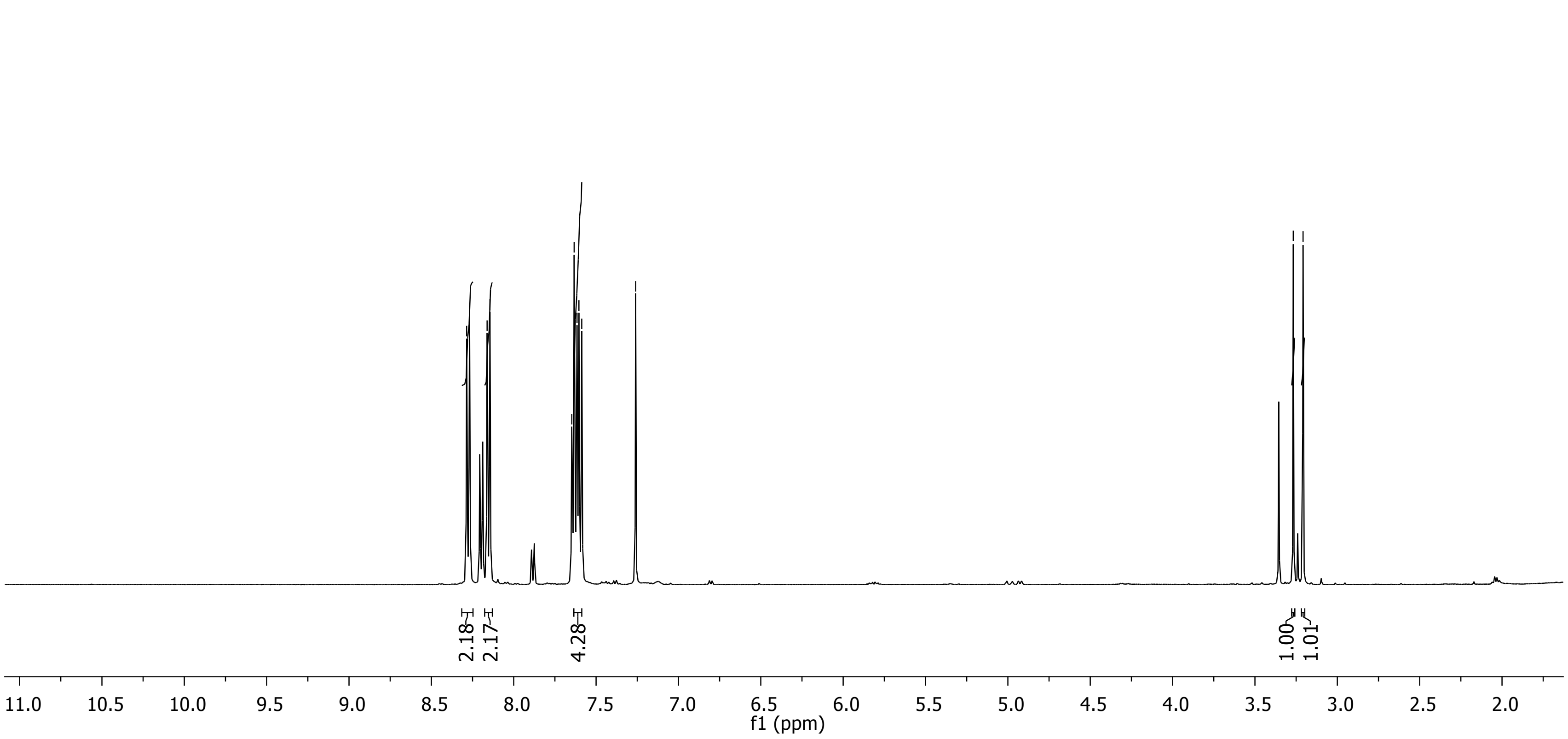

^{1}H NMR ($CDCl_3$, 500 MHz) spectrum of (*Z*)-1,2-bis(4-pentylphenyl)diazene 1-oxide (**4i**)

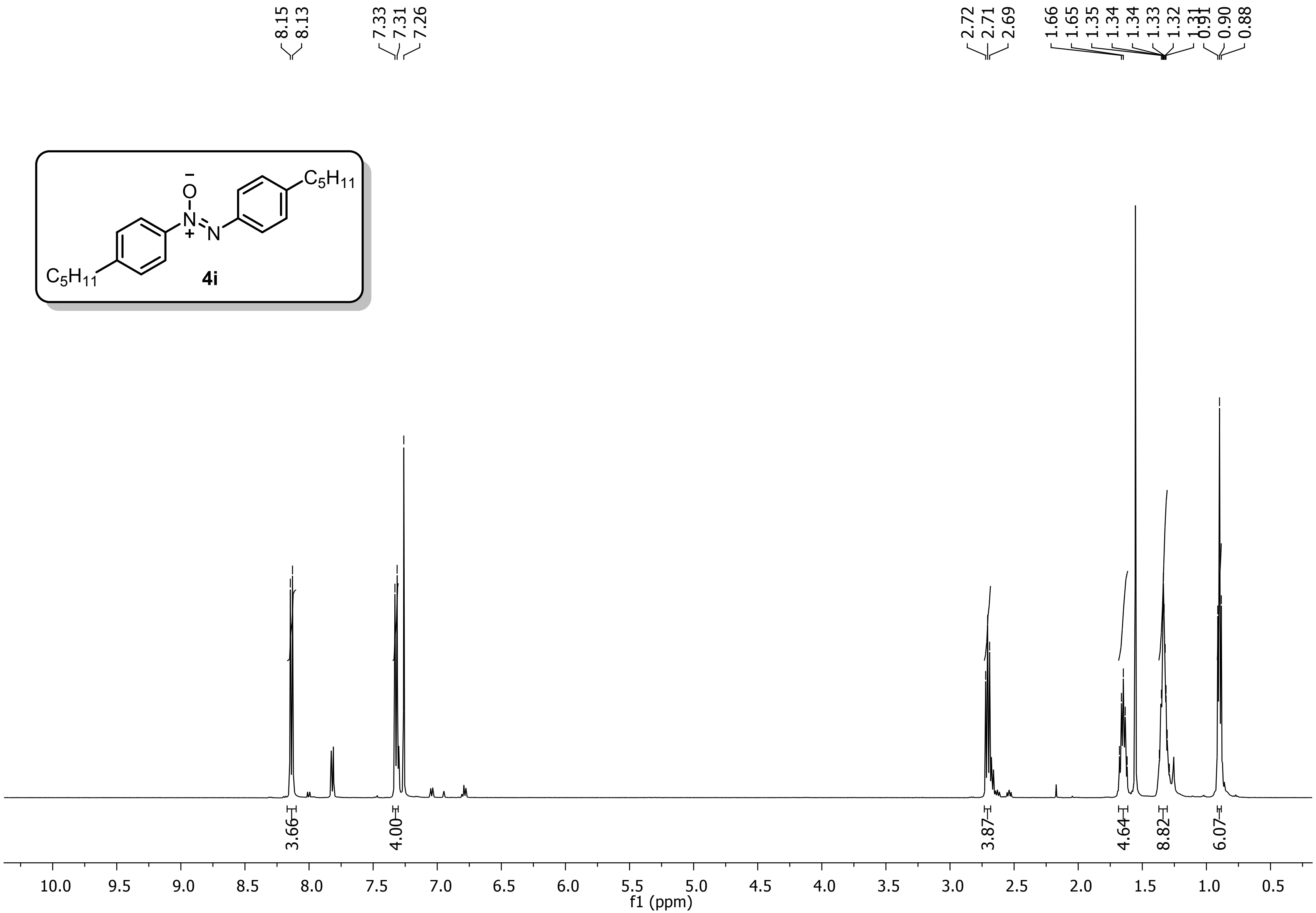

^{1}H NMR ($CDCl_3$, 500 MHz) spectrum of (*Z*)-1,2-bis(4-hydroxyphenyl)diazene 1-oxide (**4j**)

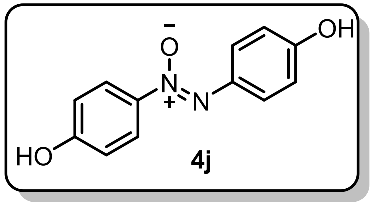

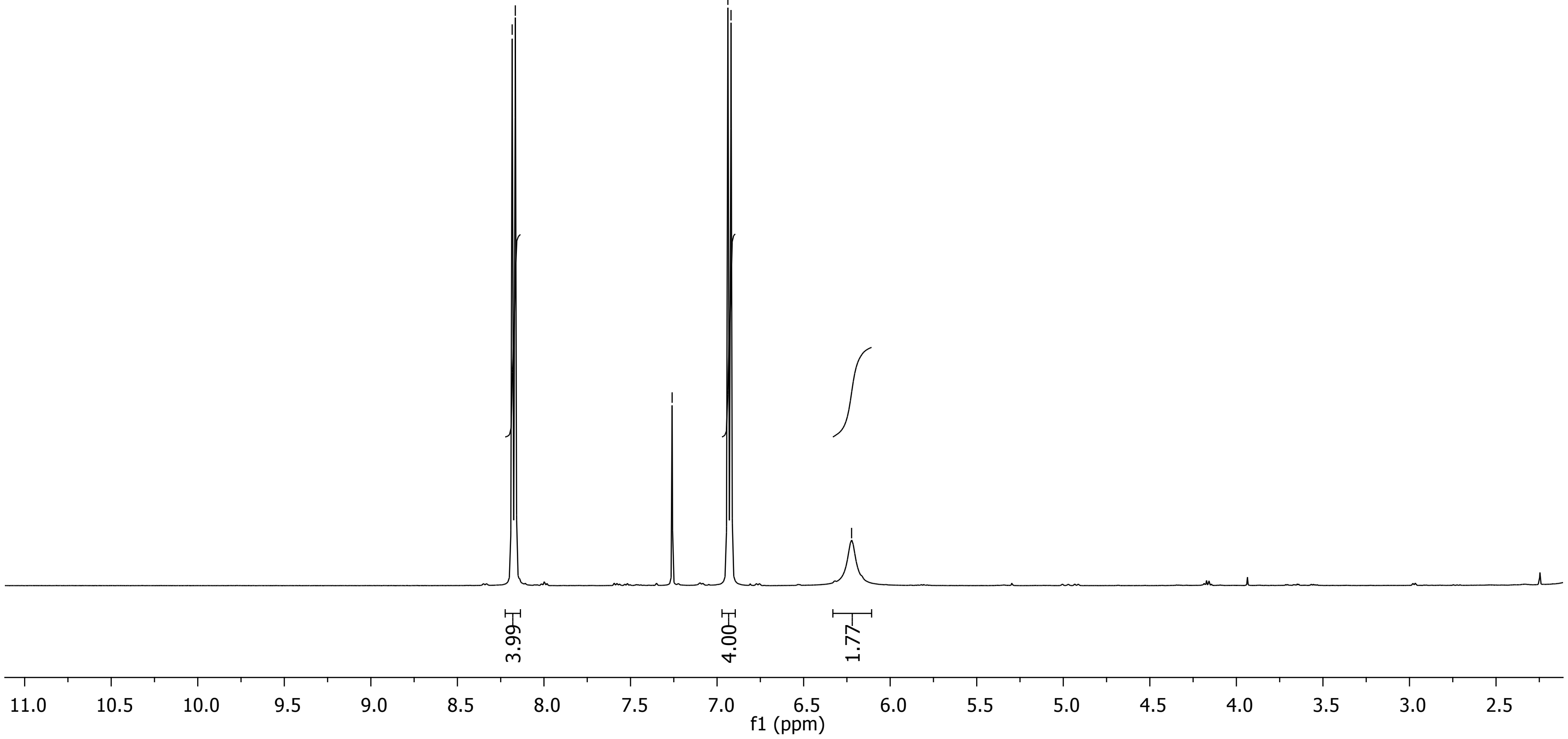

^{1}H NMR ($CDCl_3$, 500 MHz) spectrum of (*Z*)-1,2-bis(4-methoxyphenyl)diazene 1-oxide (**4k**)

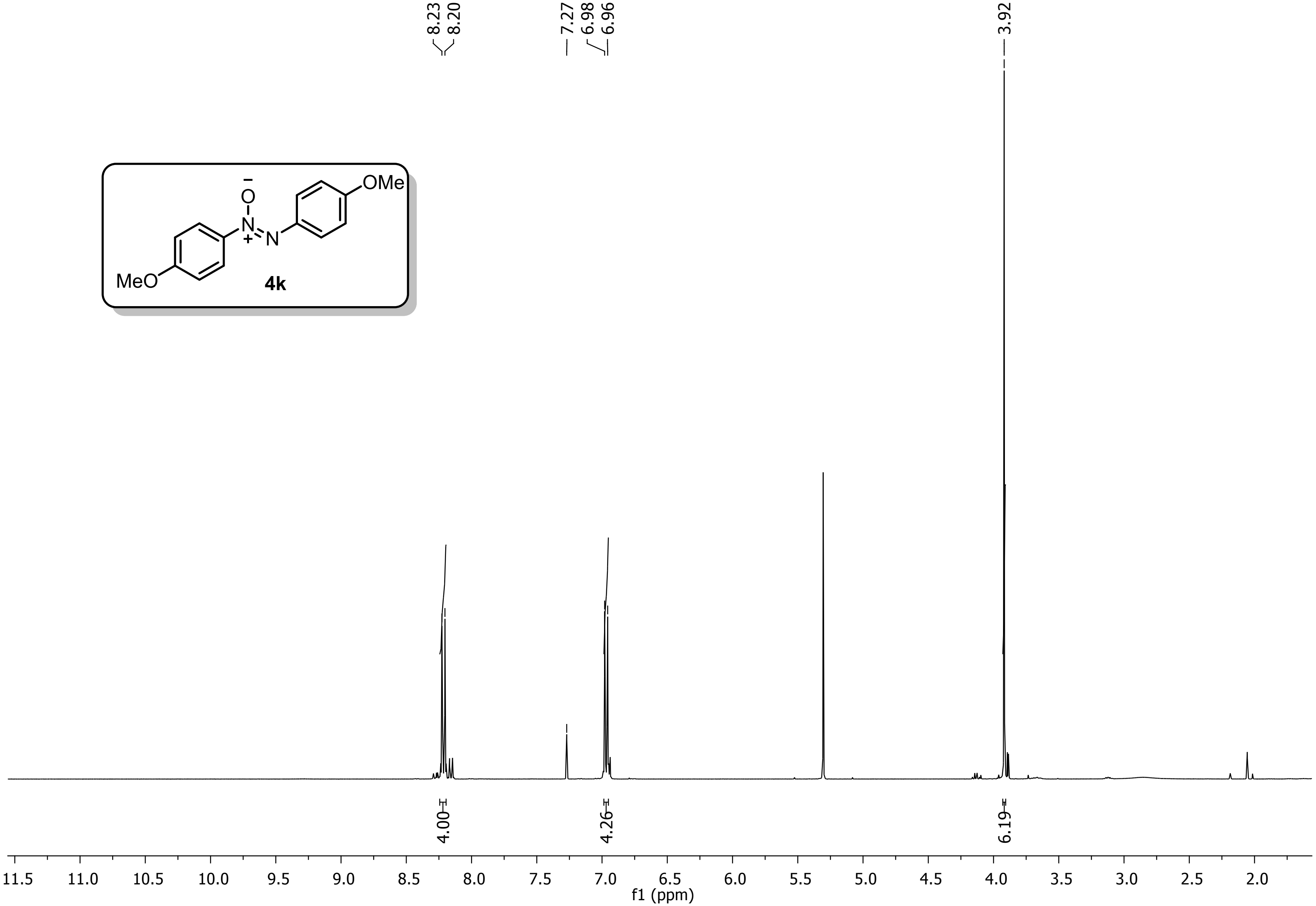

^{1}H NMR ($CDCl_3$, 500 MHz) spectrum of (Z)-1,2-bis(3-fluorophenyl)diazene 1-oxide (**4l**)

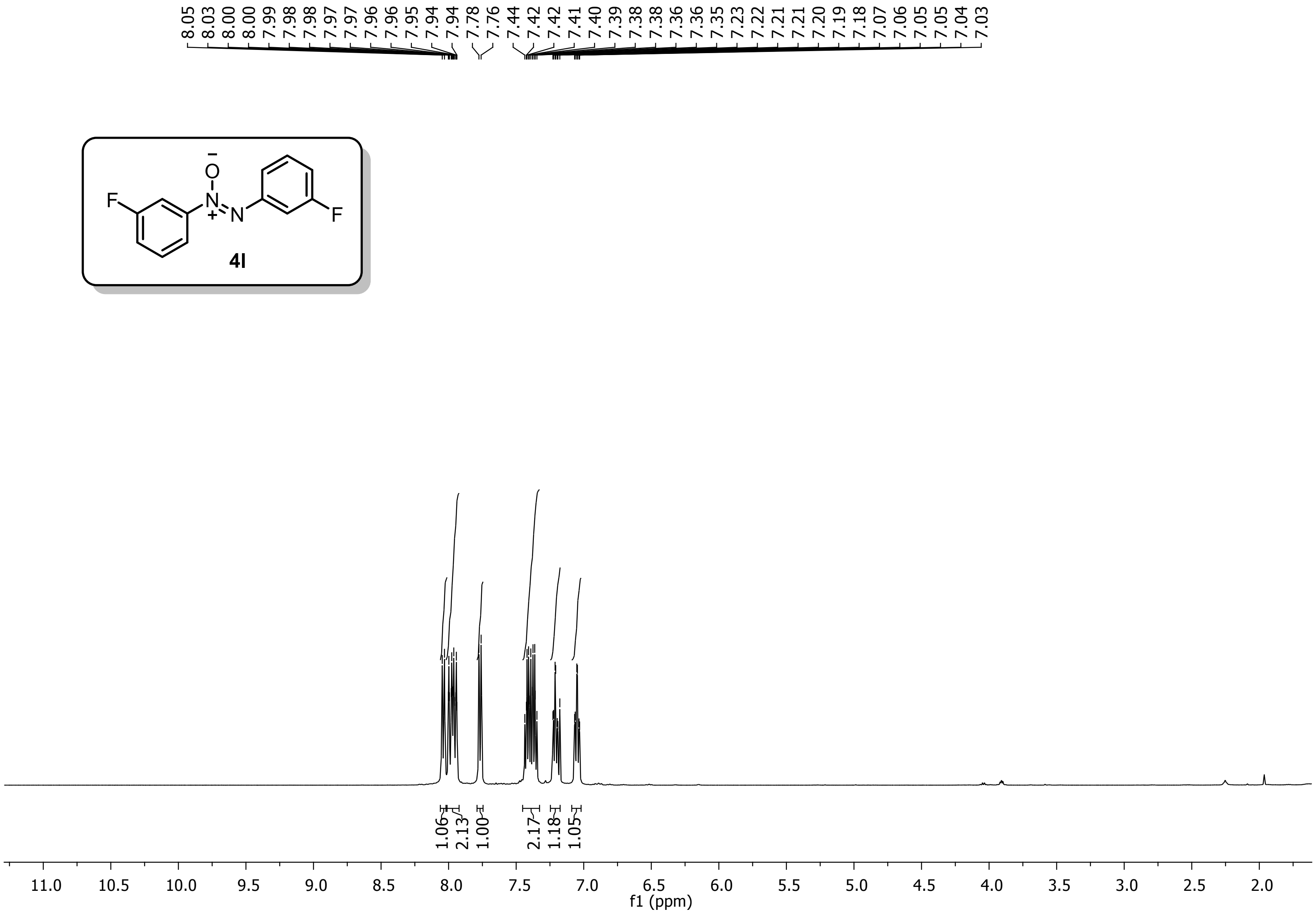

^{1}H NMR ($CDCl_3$, 500 MHz) spectrum of (*Z*)-1,2-bis(2-fluorophenyl)diazene 1-oxide (**4m**)

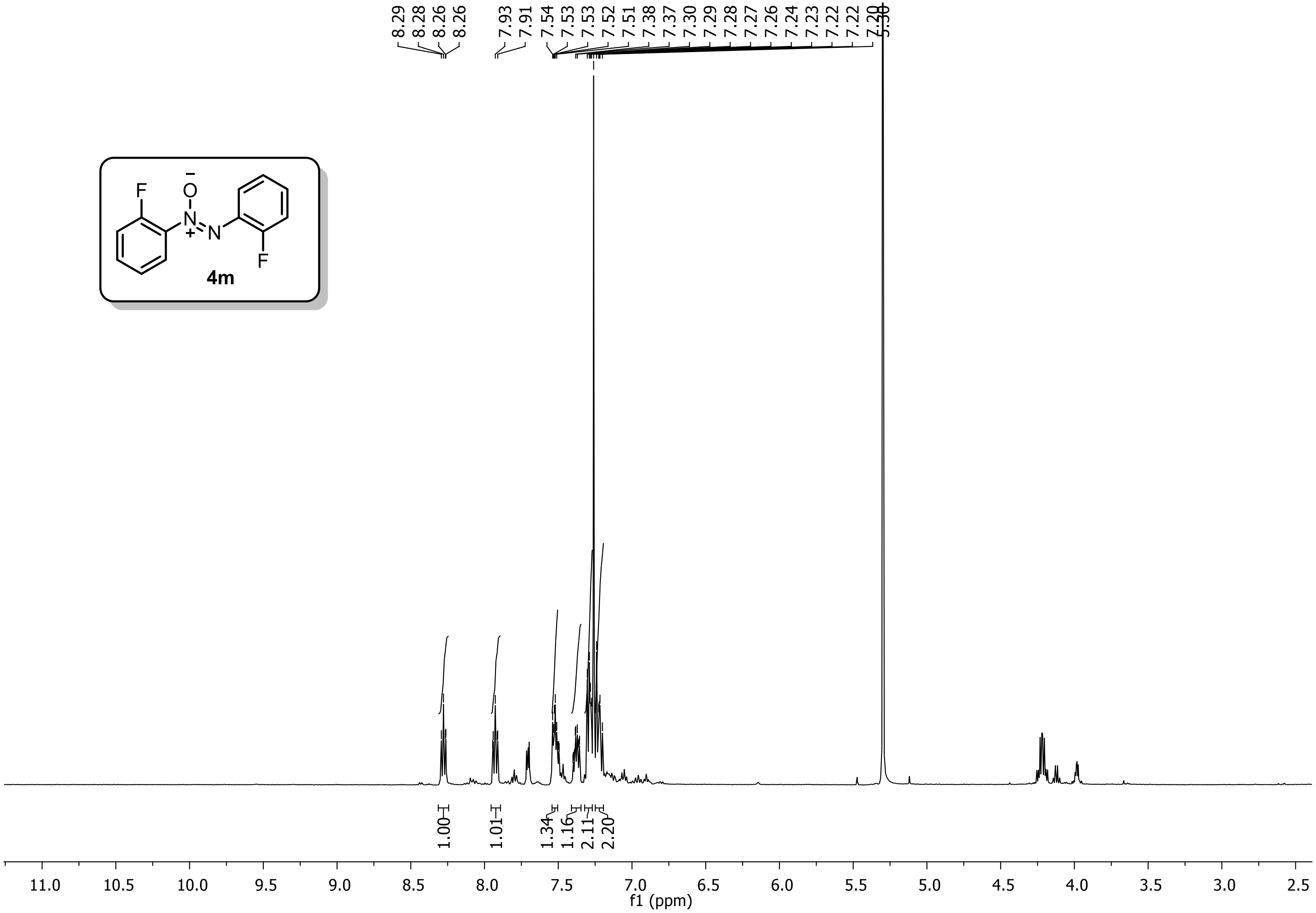

^{1}H NMR ($CDCl_3$, 500 MHz) spectrum of (*Z*)-1,2-bis(2-chlorophenyl)diazene 1-oxide (**4n**)

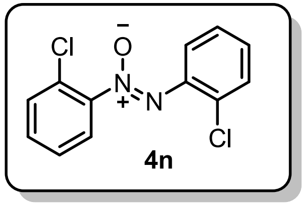

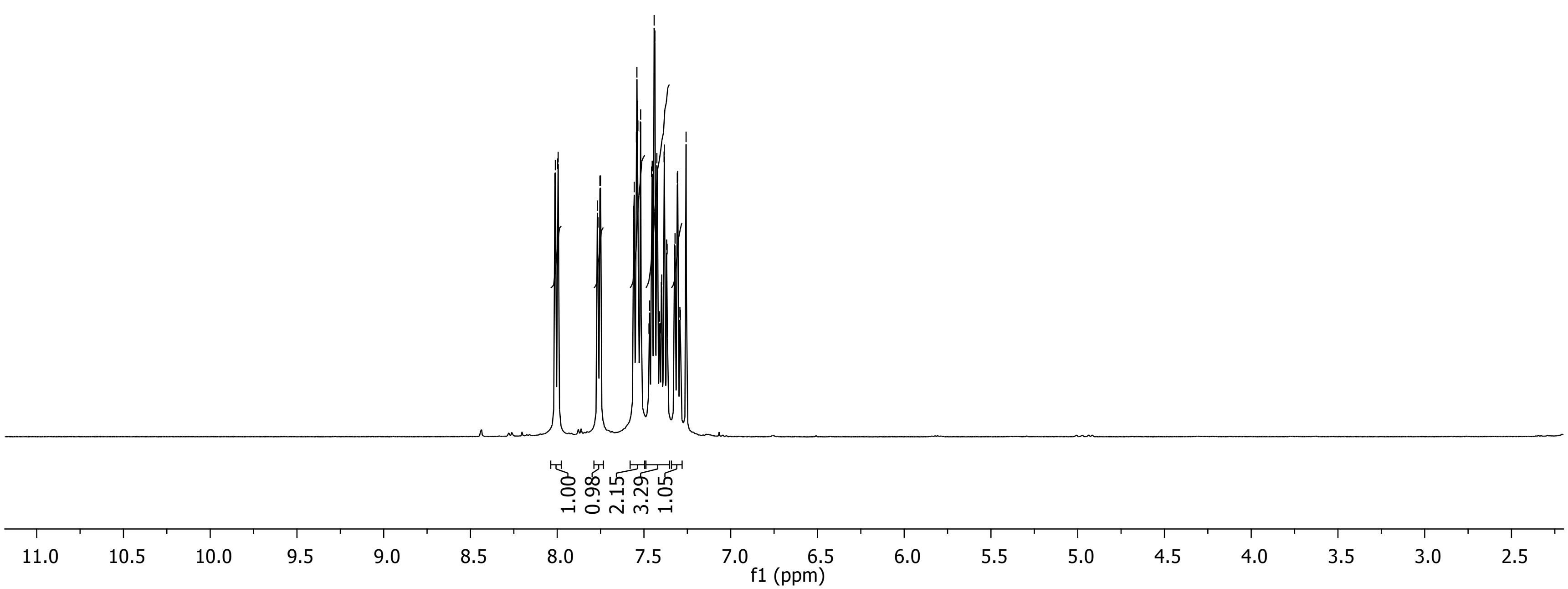

^{1}H NMR ($CDCl_3$, 500 MHz) spectrum of (*Z*)-1,2-di-*m*-tolyldiazene 1-oxide (**4o**)

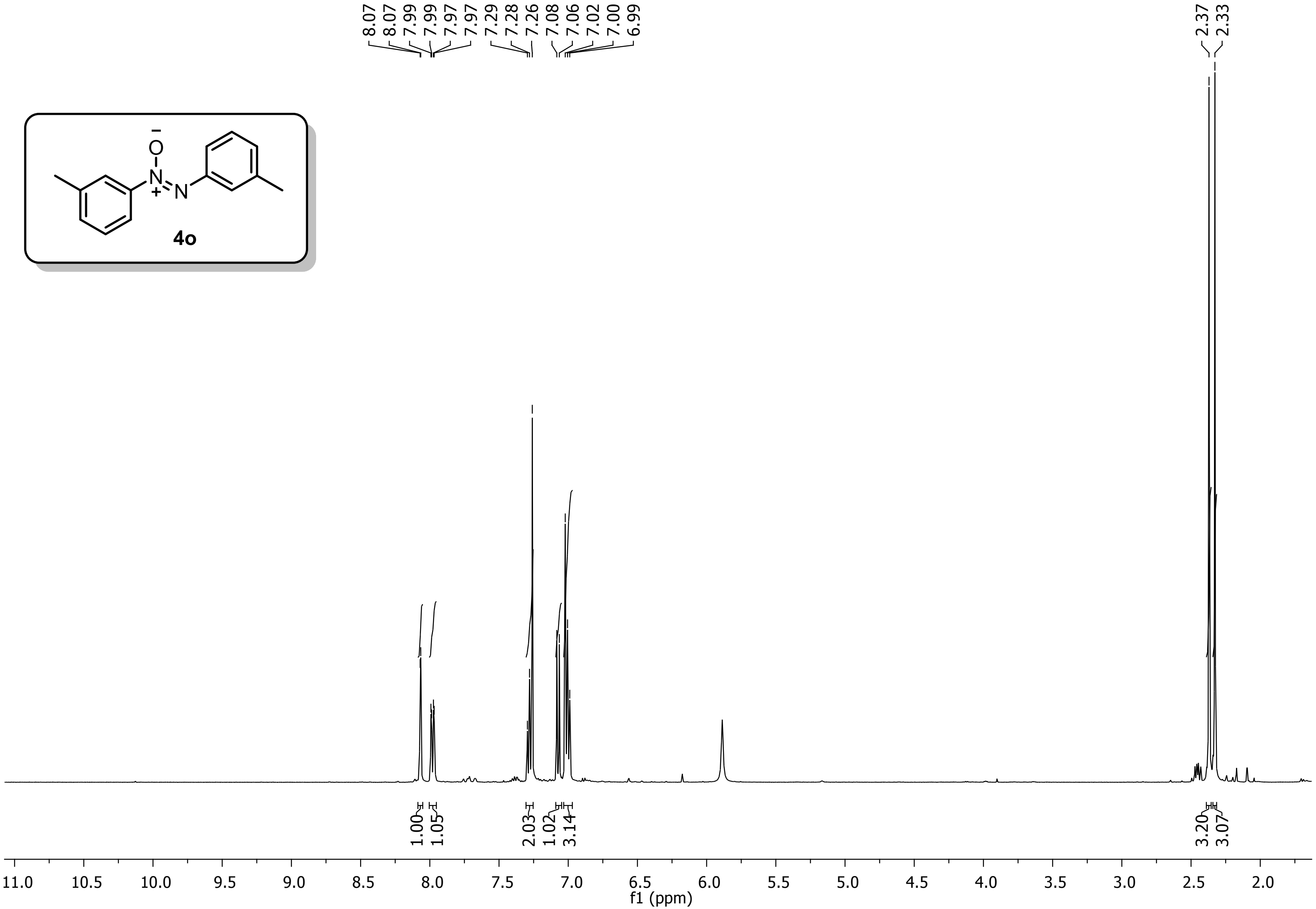

^{1}H NMR ($CDCl_3$, 500 MHz) spectrum of (*Z*)-1,2-bis(2-(trifluoromethyl)phenyl)diazene 1-oxide (**4p**)

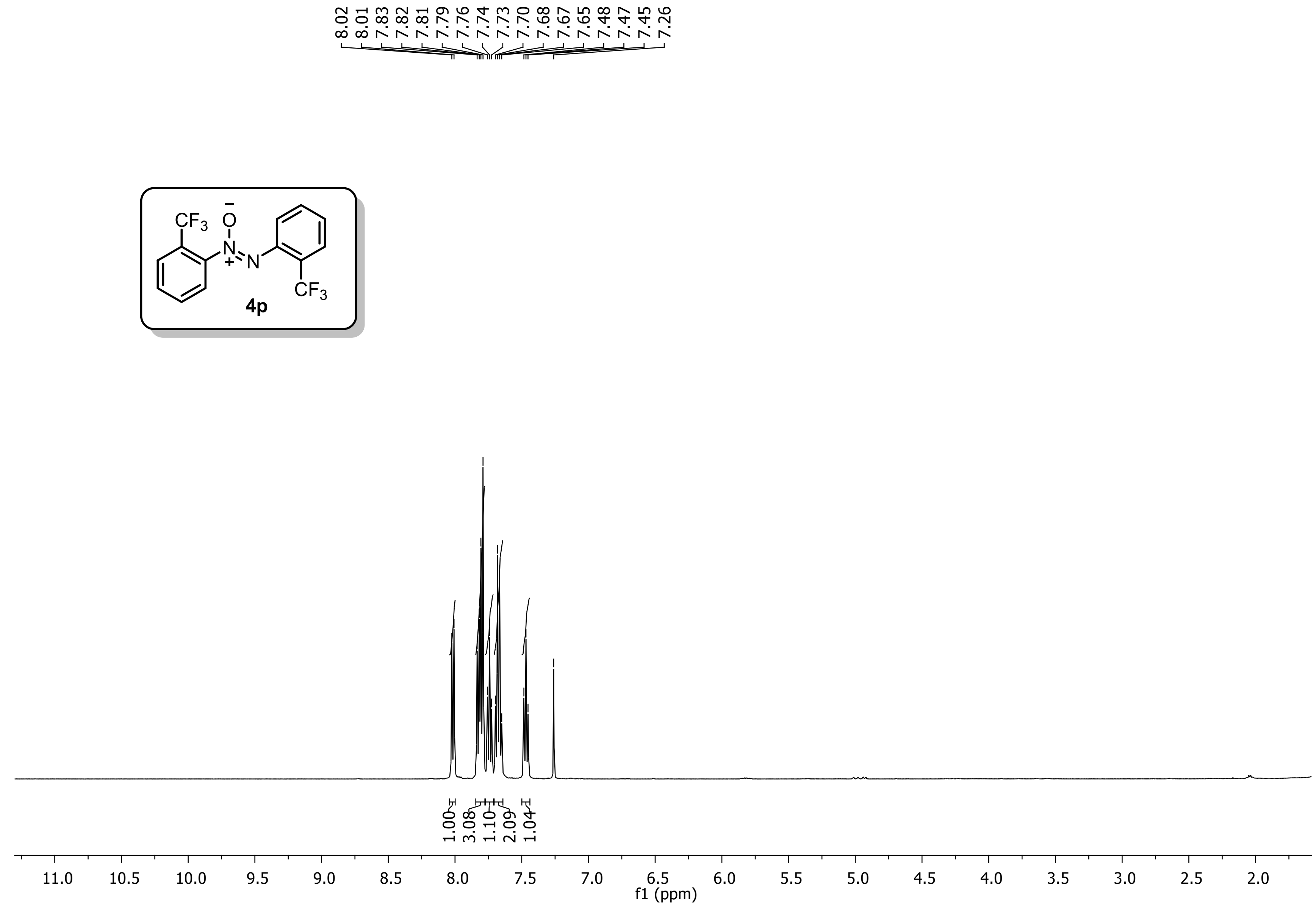

^{1}H NMR ($CDCl_3$, 500 MHz) spectrum of (*Z*)-1,2-bis(3,4-dicyanophenyl)diazene 1-oxide (**4q**)

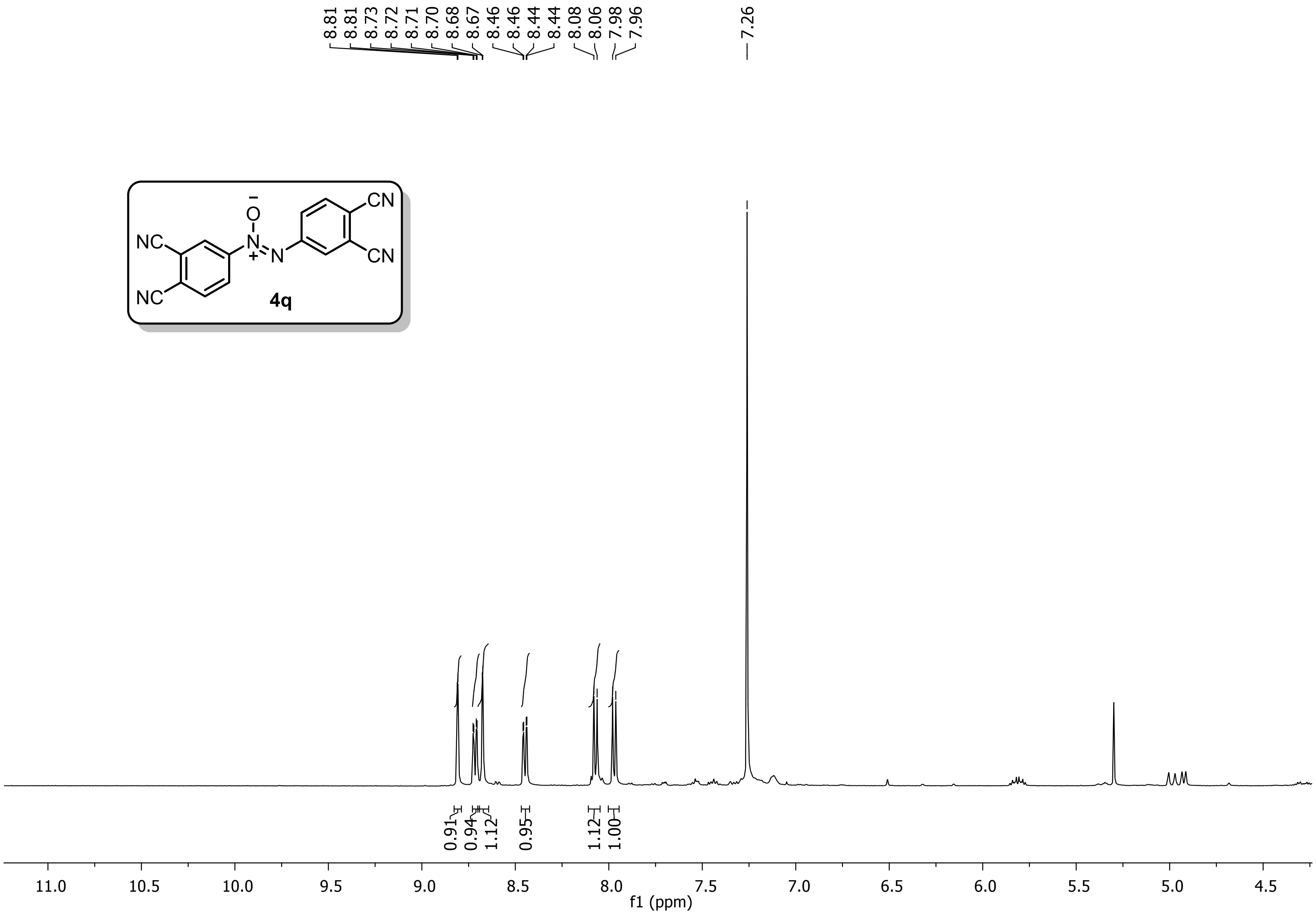

^{13}C NMR ($CDCl_3$, 121 MHz) spectrum of (*Z*)-1,2-bis(3,4-dicyanophenyl)diazene 1-oxide (**4q**)

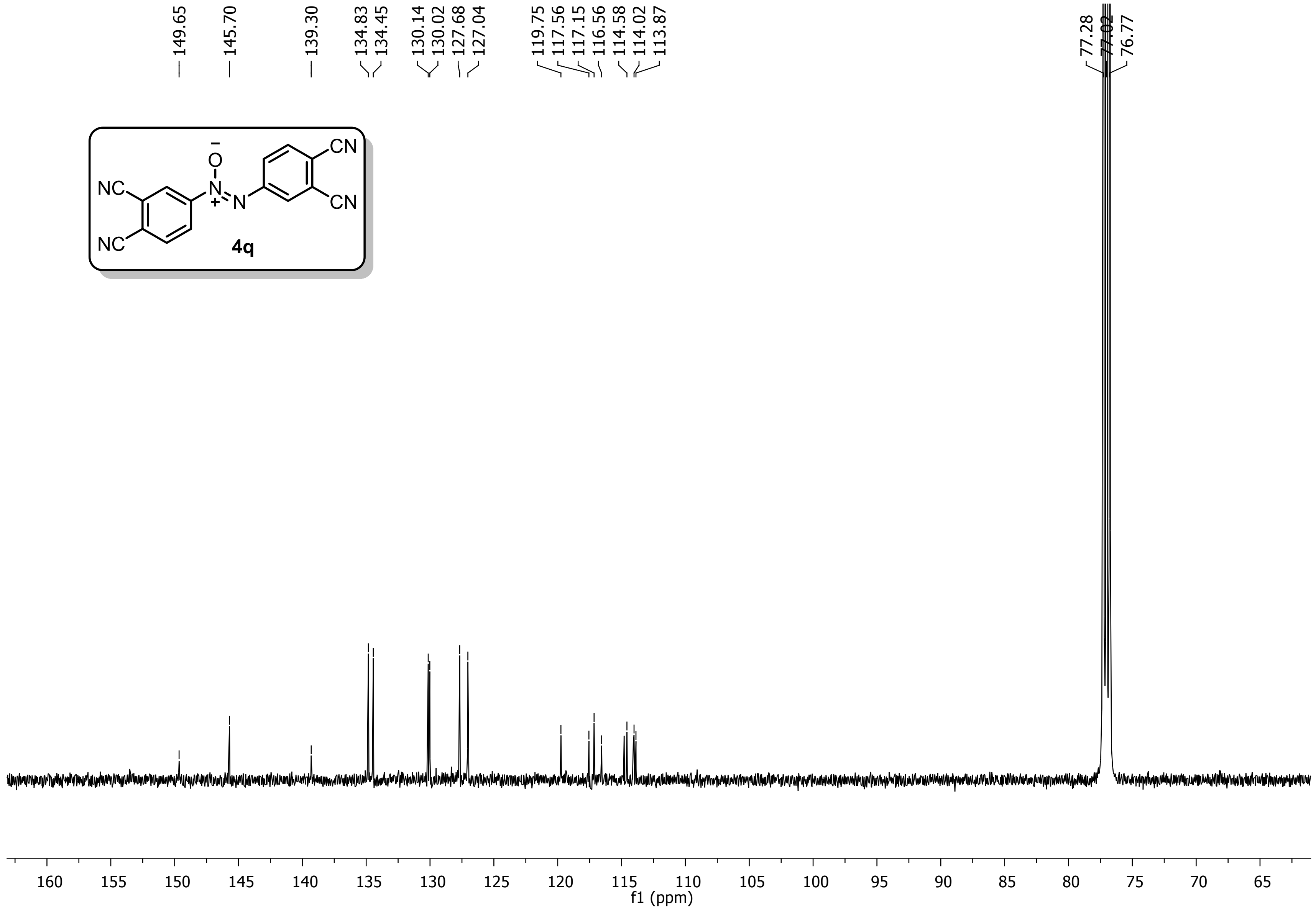

^{1}H NMR ($CDCl_3$, 500 MHz) spectrum of (*Z*)-1,2-bis(2,3,4-trifluorophenyl)diazene 1-oxide (**4r**)

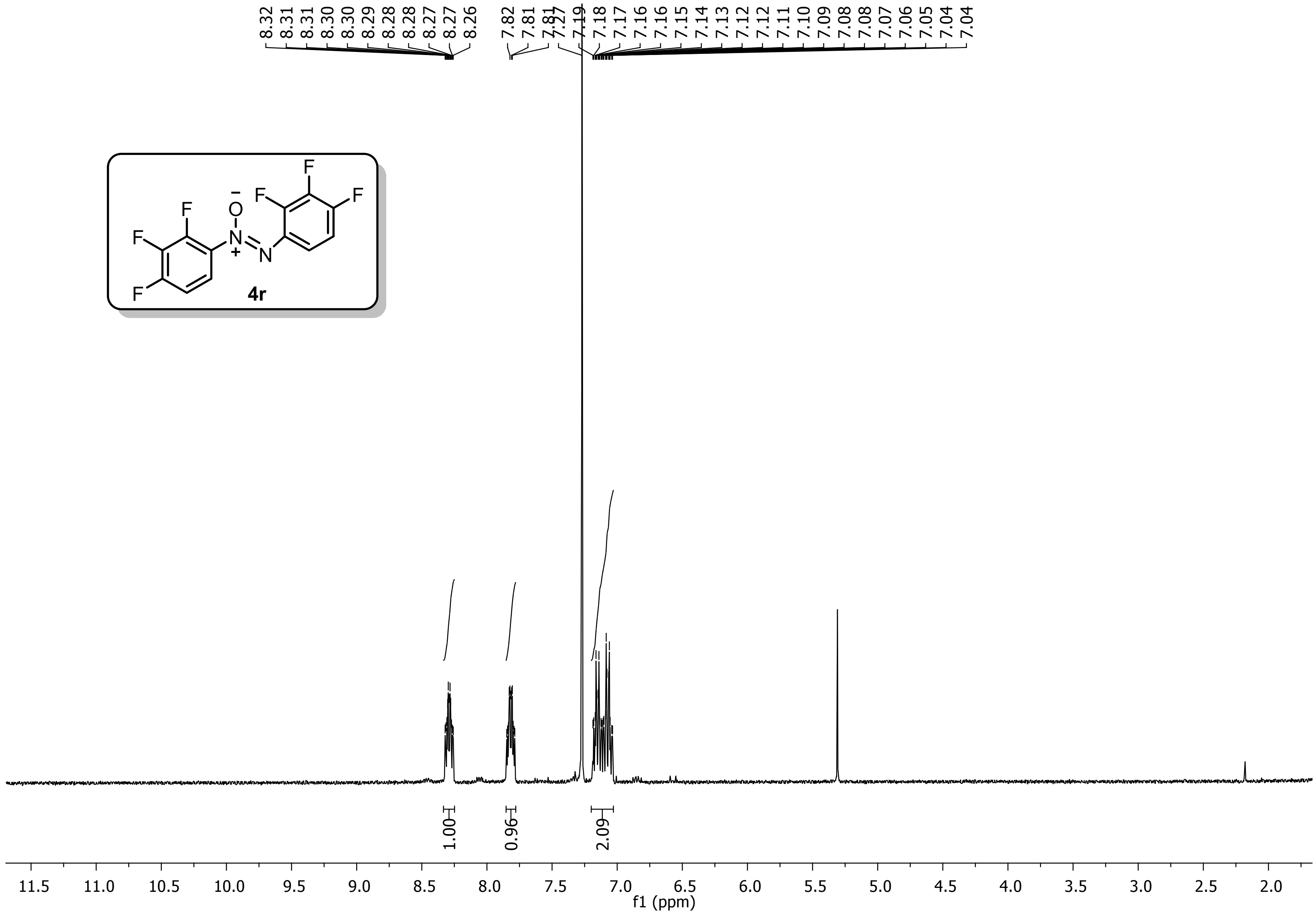

^{1}H NMR ($CDCl_3$, 500 MHz) spectrum of (*Z*)-1,2-bis(3-nitrophenyl)diazene 1-oxide (**4s**)

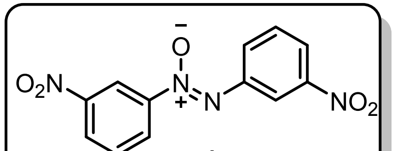

4s

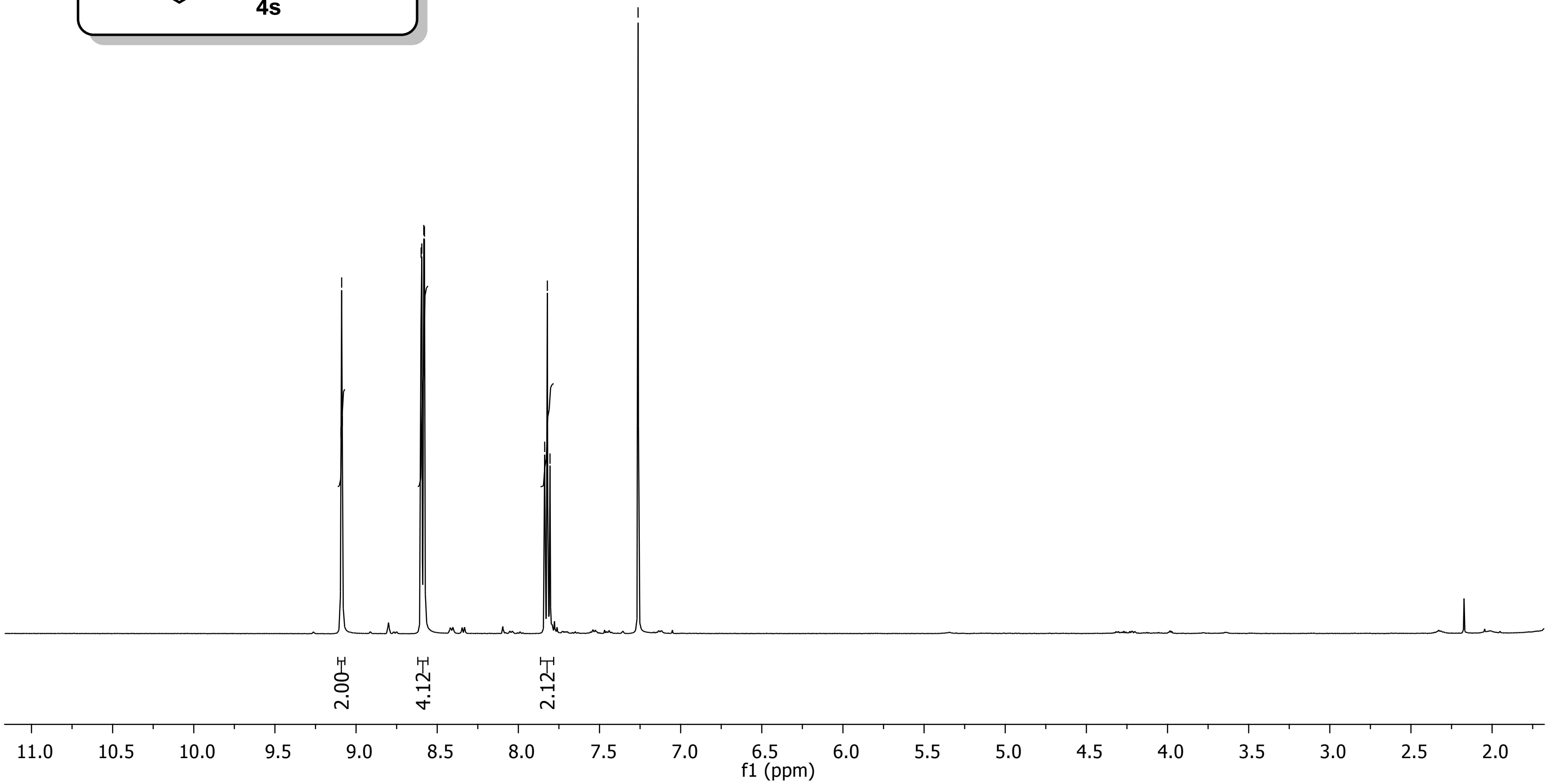

Supplement: Supplementary file 1 — ao3c08328_si_001.pdf [file ao3c08328_si_001.pdf]
